# Supplementary material for: Kinetochore protein depletion underlies cytokinesis failure and somatic polyploidization in the moss Physcomitrella patens
Source: eLife. 2019 Mar 5;8:e43652. doi: 10.7554/eLife.43652 (PMC6433463; doi:10.7554/eLife.43652)
Supplement: Supplementary file 3. [file elife-43652-supp3.pdf]

```
PpCENP-A     1 -MARRKTTPVHGNHRASTSSVGGAAVRP--------------------------------
AtCENP-A     1 -MARTKHRVTRSQPRNQTDAAGASSSQAAGPTTTPTRRGGEGGDNTQQTNPTTSPATGTR
HsCENP-A     1 MGPRRRSRKPEAPRRRSPSPT---------PTPGPSRRG---------------PSLG--

PpCENP-A    28 ---------------RKPHRWRPGTKALQEIRHYQKTCDLLIPRLPFARYVKEITMMYA-
AtCENP-A    60 RGAKRSRQAMPRGSQKKSYRYRPGTVALKEIRHFQKQTNLLIPAASFIREVRSITHMLAP
HsCENP-A    35 ------------ASSHQHSRRRQG--WLKEIRKLQKSTHLLIRKLPFSRLAREICVKFT-

PpCENP-A    72 SDVS-RWTAEALTALQEATEDYMCHLFEDTNLCAIHAKRVTIMPKDLQLARRLRGAIV--
AtCENP-A   120 PQIN-RWTAEALVALQEAAEDYLVGLFSDSMLCAIHARRVTLMRKDFELARRLGGKGRPW
HsCENP-A    80 RGVDFNWQAQALLALQEAAEAFLVHLFEDAYLLTLHAGRVTLFPKDVQLARRIRGLEEGL

PpCENP-A       -
AtCENP-A   179 -
HsCENP-A   140 G
```

```
PpKNL2-1       1 MSCQLQPGAEGSLSESVHFPPTSSFLEHQGYQGDVRMGMAPPLSSFSRAPPPRSPFLHDD
PpKNL2-2       1 ------------------------------------------------------------
AtKNL2         1 --------------------------------------MTEP------------------
HsMis18BP1     1 --------------------MIATPLKHS------RI-YLPPEASSQRRNLPMDAIFFDS

PpKNL2-1      61 GRAGSRYVHSGGFDRQGYDYAVHRHSCGDHLEL---HSGSLLEHLLLQE---NQKALRIA
PpKNL2-2       1 ------------------------------------------------------------
AtKNL2         5 ------------------------------------------------------------
HsMis18BP1    34 IPSGTLTPVKDLVKYQNSSLKLNDHKKNQFLKMTTFNNKNIFQSTMLTEATTSNSSLDIS

PpKNL2-1     115 SLVQSQASGCLSGCLSCGLGEKRLRYKDSEERMVADQRHCCPRSVGEEPSRSVNLGAEAD
PpKNL2-2       1 ------------------------------------------------------------
AtKNL2         5 -----------------------------------------------------NL-----
HsMis18BP1    94 A-IKPNKDGLKNKANYESPGKIFLRMK---EKVLRDKQ--------EQPSRNSSL---LE

PpKNL2-1     175 PVECADEWFRQPRP----------------------------RESVGGV-----------
PpKNL2-2       1 ------------------------------------------------------------
AtKNL2         7 ------------------------------------------------------------
HsMis18BP1   139 PQKSGNNETFTPNRVEKKKLQHTYLCEEKENNKSFQSDDSSLRASVQGVPLESSNNDIFL

PpKNL2-1     196 --------GERSQAKISFFEDELGRSLCQERRTVFPVS--RPVLSHSQASHHIHDHQDHL
PpKNL2-2       1 -----------------------------------------------------MLDQQPH-
AtKNL2         7 ------------------------------------------------------------
HsMis18BP1   199 PVKQKIQCQQEKKAPLHNLTYEL-PTLNQEQENFLAVEARNKTLTRAQLAKQIFHSKESI

PpKNL2-1     246 VGAQESSVPIYGFSDGDGRVRQRWPSQRPAYS--------GNGVEVDGWQRATRSVS--R
PpKNL2-2       8 VGAQESSAPMNGFSDADARVRQRWSCQRQAYS--------GDGAEENCWQRATKSVS--R
AtKNL2         7 --------------DEDGS-----------------------------------------
HsMis18BP1   258 VATTKSKKDTFVLESVDSADEQFQNTNAETLSTNCIPIKNGSLLMVSDSERTTEGTSQQK

PpKNL2-1     296 VGSVT-ATEQDRLSLPDIAAGSLPTVLVGSPHRPVGDASRPTQSARTTSQNLLRM-SNSV
PpKNL2-2      58 VGPAN-PRLQDRVSLSGRATGLLPTALAETPHRPVVDSGRSTQSTHTASQNPLGIPENSL
AtKNL2        12 --------------------------------------------------------KSSF
HsMis18BP1   318 VKEGNGKTVPGETGLPGSMKDTCKIVLA-TPRLHITIPRRSKRN------------ISKL

PpKNL2-1     354 EPSSRDKPLQEASTQRVVTQGPVSLTGWYIMKVQTADVGRVVETKVAVGGRLVQGGEHVK
PpKNL2-2     117 EPSYQDNAVQEAATERLITQGPVSLTGWYIIKVEITNVGRVVETKIAVGGRLLQNGEHVK
AtKNL2        16 -------------------QKTVVLRDWWLIKCPKEFEGK----QFGVAGF----EESVE
HsMis18BP1   365 SPPR----IFQTVTNGLKKNQVVQLQEWMIKSINN-------NTAICVEGKLID----VT

PpKNL2-1     414 TS--------PIVNRLDFHKVVTEDGVEVSLEGSMDLETSTANGFSPGIVQCLCNGFPYM
PpKNL2-2     177 TR--------CIVNRVDFHQVVTEDDIEVSLEGSMDLGTSIANGFAPGIVQCLSNGFPYM
AtKNL2        49 TRAMRVFTSSPITKALDVFTLLASDGIYITLRGFLNKERVLKNGFNPEISREFIFGFPPC
HsMis18BP1   410 NI---YWHSNVIIERIEHNKLRTISGNVYILKGMIDQISMKEAGYPNYLIRKFMFGFPEN

PpKNL2-1     466 WKQLL-----RVRPVGMSSSLAVSESVGGLHPVASKCVSEDESQGVVDPNICKTIPKGIP
PpKNL2-2     229 WKQLL-----RVRTVGLSSSLAVSESIGGLHPEASKCASEDESQGFVDLKTSGVIPKDIS
AtKNL2       109 WERV--------------------------------CNS---------------------
HsMis18BP1   467 WKEHIDNFLEQLR--------------------AGEKNREKTKQKQKTGRSVRDIRKSMK

PpKNL2-1     521 GGSRDC-EGNSTVGDVGSDAVAAVDEPSVIQRIASDAVGSKRVETEAVVTAGAETSTAVE
PpKNL2-2     284 NSLKHC-ESNSPVAGVN-------EEVSVC--ITSDGVETKGVETEAVVVTWAEASNAFD
AtKNL2       116 -----CFEGDSFGTDVN-------------------------------------------
HsMis18BP1   507 N------DARENQTDTA-------QRATTTYDFDCDNLELK--------------SNKHS

PpKNL2-1     580 TEPVDPGGV-----------------------ERCEPPNAFEIEAMILGGGTTVRDEPPN
PpKNL2-2     334 SEPTELEGLVSASFETSKLVGMGAMNSKVAASARFDAANAVGREVVEPGGAATVRAEMSN
AtKNL2       128 TVPSTIEKA---------------------------------------------------
HsMis18BP1   540 ESP----------------------------------------------GATELNMCHSN
```

```
PpKNL2-1    617 ADGKGTVEPEGEGIGRANTSNAIDEGCPLDVPSQQACDPCPTNFDPVPVSNPLDTDSLGK
PpKNL2-2    394 SVEREAVERERVATARTISSNAIDESNSHDITR-------PTSWDFVPGNEAPVSESLSK
AtKNL2      137 ---------------------------------------CPPILSPCKYSNRNLKDNPAE
HsMis18BP1  554 CQNKPTLRFPDDQVNNTIQNGGGDDLSNQELIGKKEYKMSSKKLKIGERTNERIIKSQKQ

PpKNL2-1    677 EAESGPTVDTTFVTMRR-----------GRKNKKG--PPQPVRSSARLQQRRTKSAESMP
PpKNL2-2    447 EAESGSVLDTAFVTARR-----------GRKNKKG--SAQPVRSSRRHQQQQSRSTKLLQ
AtKNL2      158 SREKSNVTETDIAEI----------------NDKGGSGARDIKTARR------RSLHL--
HsMis18BP1  614 ETTEELDVSIDILTSREQFFSDEERKYMAINQKKAYILVTPLKSRKVIEQ---RCMRY--

PpKNL2-1    724 NLFKSSGPAQVEQVTVSGEVTRDNSGEQELGEIILPDVQRAANGREKITRTSDIIG----E
PpKNL2-2    494 TLVDSSGTAQVEQFTSGVEVTKEMFGEEEPRKVALDFEQDATGSKELTTINSIIE----E
AtKNL2      194 ---------QIKRILESSKVRK---------------------------TAND--------
HsMis18BP1  669 NL--SAG--TIKAVTD---------------FVIPECQKKSPISKSMGTLENTFEGHKSK

PpKNL2-1    780 VLKDLQEEDMEAANQGGVPGAGFVGIVVDPQPIQVEISVQVQDSGKNLLKDRDDVE--VP
PpKNL2-2    550 VLNDLQQEDFETINQGGDLKAGCSSISAQRQPTEMKKSGQVQDVAVNLLKDRYGVE--AL
AtKNL2      211 --GDHGSEFLNTAKRGDVERDGCEVINNEDSEWKLDES-EVQ----NLCNDGDNGSEGFI
HsMis18BP1  710 NKEDCDERDLLTVNR-----------------KIKIS---------NLEKE---------

PpKNL2-1    838 KLNENDATEDRMEIEENKEMAPAASSDFE---NEKSEETAQAAPAATFKLVNEEMEE--A
PpKNL2-2    608 KLNEKGATEDVLESAKDKETTSAALLELE---SEKIEEVTPAAPS---KLGSEEMEE--S
AtKNL2      264 KAKSSDVEKDKSEAIDNDVISPAVGSGIKHTGADNVDKVTSASATGE-SLTSEQQNGLLV
HsMis18BP1  735 QMLTSDFKKNTRLLPKLKKIENQVAMSFYKH---------QSSP----DLSSEESET---

PpKNL2-1    893 APVTPPELIQRKVQSRVDSRYKPSSEALPQASEERVSPELNARMTRSLKRRLRLSAISEP
PpKNL2-2    660 APVTPPELLLSKVQSRVHFHDKLSFEALPQASEELLFPEVNVRITRSLKRRLRQPAITD-
AtKNL2      323 TTASPHSLLKDLAKSS-----KPEKKGISKKSGKILRSDDN---------------VVDP
HsMis18BP1  779 -----EKEIKRKAEVK-----KTKAGNTKEAVVHLRKSTRN---TSNI------PVILEP

PpKNL2-1    953 VADVAPVTRSSKRLRRPVPESNSEPSISHQVGDPL-------VQPVSNPDLSHQ------
PpKNL2-2    719 -------AKRSRRLSK--PEGQSAKSVTY-LSSPL--EEGPSTEPNSNPSASHH------
AtKNL2      363 MNYSGTKVKSAENKRK--IDASKLQSPTSNVAEHS--KEGL------NNAKSND------
HsMis18BP1  820 ------ETEESENEFY-IKQKKARPSVKETLQKSGVRKEFPITEAVGSDKTNRHPLECLP

PpKNL2-1   1000 ------------VEGPSMEPISNPV-TPHHGEVNGFHARV-------------------
PpKNL2-2    761 -------------ENLTLEPISDPA-TPLQEAVNEFYLRG-------------------
AtKNL2      407 ------------VEKDVCVAINNEVISP----VKGF-----------------------
HsMis18BP1  873 GLIQDKEWNEKELQKLHCAFASLPKHKP------GFWSEVAAAVGSRSPEECQRKYMENP

PpKNL2-1   1027 ------RDLSTRRAKAVNCSNCKKPCSSQ-EVIKYKNRIASIGATPEHA--VERTMKTEQ
PpKNL2-2    787 ------RHPYSSSAKVSRCSNCKRPCSPQ-AMVDYKTSVVSSKATTELV--VERALKMEQ
AtKNL2      427 --------------GKRLSGTDVERLTSKN-AT---KESLTSVQRKGRVK--VSKAFQ---
HsMis18BP1  927 RGKGSQKHVTKKKPANSKGQNGKRGDADQKQTIKITAKVGTLKRKQQMREFLEQLPKDDH

PpKNL2-1   1078 EE---YPPLEPQR------TESTPGTSSLPKKGETNTSSAKRRSGRPKKTKS--------
PpKNL2-2    838 ED---CSPLEFQN------PAKTCGTTSLPDKDKSGTSSGKRRSHHPKKVKS--------
AtKNL2      465 -------DPL---------------------SKGKSKKSE---------KTLQS--------
HsMis18BP1  987 DDFFSTTPLQHQRILLPSFQDSEDDDDILPNMDKNPTTPSSVIFPLVKTPQCQHVSPGML

PpKNL2-1   1121 ---ANLPAERRTTRRLKLDDSGEGPSEGHNPVPSKQSSRRTSIMPPPLPSPRV-QGGLKG
PpKNL2-2    881 ---ASQPAKGTMTRRSKLDDSGEGSSAGCNPVSSKRNNRRTSIIPPPLPPPRV-ENGLKS
AtKNL2      482 ---NSNVVEPMNHFRSEAEEAEEN-------LSWEKIKRKIDFDVEVTPEKKVKQQKTNA
HsMis18BP1 1047 GSINRNDCDKYVFRMQKYHKSNGG-------IVWGNIKKKLVETDFSTPTPRR-KTPFNT

PpKNL2-1   1177 KVPEAFGLKTSRSGRLLVPALAYWRSQSIEY---DKDGGIIAIFDGFQATPSDTGCFNFT
PpKNL2-2    937 KVPEAFGLKTSRSGRLLVPPLAYWRSQTIEY---DKDGGIIAIFDGFQ------------
AtKNL2      532 ASTDSLGQKRSRSGRVLVSSLEFWRNQIPVY---DMDRNLIQVKDGSE---------TNSA
HsMis18BP1 1099 DLGENSGI-----GKLFTNAVESLDEEEKDYYFSNSDSA---------------------

PpKNL2-1   1234 PPQEKHAKKIQEKLCKAAATVKKRK-----
PpKNL2-2        ------------------------------
AtKNL2      581 PSKGKGSDS------------RKRRNLKIK
HsMis18BP1      ------------------------------
```

```
PpCENP-C    1 MRGGMVGFVVNEKENTPVREKGKKGTGTKSKEAEISRRLSISESKSRPHYLRVIT--NRF
AtCENP-C    1 -------------------------------MADVSRSSSLYTEEDP---LQAYSGLSLF
HsCENP-C    1 ----MAASGLDHLKN-----------GYRRRFCRPSRARDINTEQGQ-NVLEILQ--DCF

PpCENP-C   59 QR--KGGSGRGRITAHRPIHLDGSPYEFSSRSDR----EGNKRKSFTVYEDDMDCMSCSF
AtCENP-C   27 PRTLKSLSN--------PL-----PPSYQS--------EDLQQTHTLLQSMPFEI-----
HsCENP-C   43 EE--KSLAN-----------------DFSTNSTKSVPNSTRKIKDTCIQSPSKEC-----

PpCENP-C  113 KQPKFPIELGSSRNVPTVAGSKNATLERC--------RTLNDGGKWEQIAPTLVGQQE-P
AtCENP-C   61 -QSEHQ-------------EQAKAILEDV-----------------------DVDVQLNP
HsCENP-C   79 -QKSHP------KSVPVSSKKKEASLQFVVEPSEATNRSVQAHEVHQKILATDVSSKNTP

PpCENP-C  164 KLHILESFTAFRARNKARFSVNL---KPSLLEPANQQESGSVSLEVLAA---------RW
AtCENP-C   84 IPNKRERRPGLDRKRKS-FSLHLTTSQPPPVAPSFDPSKYPRSEDFFAAYDKFELANREW
HsCENP-C  132 DSKKISSRNINDHHSEADEEFYLSVGSPSVLLDAKT----SVSQNVIPS---------SA

PpCENP-C  212 QRGGRRSLYT-RRSLGM---SELEDIKE--------------SFR-KFEEQILEKEMHDQ
AtCENP-C  143 Q---KQT------------GSSVIDIQENPPSRRPRRPGIPGRKRRPFKESFT-----DS
HsCENP-C  179 Q---KRETYTFENSVNMLPSSTEVSVKT--------------KKRLNFDDKVMLKKI---

PpCENP-C  253 YLEKEISNQHVEKEI---HDQYLEE------EMHDQYLDEEMREQDFERENHEQD----V
AtCENP-C  183 YFTDVINLEASEKEIPIASEQSLESATAAHVTTVDREVDDSTVDTDKDLNNVLKDLLACS
HsCENP-C  219 ----EIDNKVSDEE--------------------DKTSEGQERKPSGSSQNRIRD----S

PpCENP-C  300 EKKMHDQDTEKEMHDQDVEKEVNDQDAEKE---MLEENLLDQGRSI----VP-VQDSVAR
AtCENP-C  243 REELEGD------------------GAIK----LLEERLQIKSFNIEKFSIPEFQD--VR
HsCENP-C  251 EYEIQRQ-------------------AKKSFSTLFLETVKRKSES----------SPIVR

PpCENP-C  352 EAAAVPVDA----------QENVRESGPEPFCSLDWELSSPRSGGLETEDETAYVDLSTP
AtCENP-C  279 KMNLKASGSNPPNRKSLSDIQNILKGTNRVAVRKNSHSPSPQTIK----------HFSSP
HsCENP-C  282 HAATAPPHSCPPDDTKLIEDEFIIDESDQSFASRSW-ITIPRKAG------------SLK

PpCENP-C  402 QLNVSKDKLRLNQEALFSESTSVIL------ELGVSPLASDSNEAIEQLTPANLPT--TG
AtCENP-C  329 NPPVDQ--------FSFPDIHNLLPGDQQPSEVNVQPIAKD----IPNTSPTNVGTVDVA
HsCENP-C  329 QRTISP-----------AESTALL-------------QGRKSREKHHNILPKTL------

PpCENP-C  454 CSADDTPQQSLEELESELAQTVEAVQVWSVELDPSRRRRESEYYNKRHSPGLRRFVLSSS
AtCENP-C  377 SPFNDSVVKRSGEDDSHIHSGIHRSH-------LSRDG----------NPDIC--VMDS-
HsCENP-C  359 --ANDK--------HSHKPHPVETSQ-------PSDKT-----------------VLDTS

PpCENP-C  514 KLANQVPET-TDYVMPTPTMFSDHVDRATQFNDGWPSSMTPNEFITSKPVNEPVQSCEGG
AtCENP-C  417 -----ISNR-------SSAMLQKNVDMRTK-----------------------------G
HsCENP-C  385 YAL--IGETVNNYRSTKYEMYSKNAEKPSR------SKRTIKQKQRRKFMAKPAE-----

PpCENP-C  573 LQSTDLPSSEKSLNEVPRDVMITASGFPSCLDHAAPDELETQDSFTDNE----RETQAWG
AtCENP-C  436 -KEVDVPMSESGAN---RN---------------------TGDR--END----AEINEET
HsCENP-C  432 -EQLDVGQS--------KDENIHTSHI-------------TQDEFQRNSDRNMEEHEEMG

PpCENP-C  629 DSKTTPTTCGSPWPDQSPVPPM------------RSSAQPSR--LHEKAKSSFGSPSLQL
AtCENP-C  465 DNLERLAECAS----KEVTRPFTVEEDSIPYQQGASSKSPNR------------------
HsCENP-C  470 ND------CVS----KKQMPPV------------GSKKSSTRKDKEESKKKRFSSESKNK

PpCENP-C  675 EVPRRDDSMAATPLMLKPSFDTFGMSLTPPRNPCLLFGDPFMRQMNGEELSRMWLQFCTS
AtCENP-C  503 -APEQ--------------YNTMGGSLEHAEHNQGLH---------------------EE
HsCENP-C  508 LVPEEVTS-------------TVTKSRRISRRP-----------------SDWWVVKSEE

PpCENP-C  735 PTVVSDGLVG--KKNSSTVESPSTRPICRQPRASFQSEQVLQRTTKVDVVPRTDEEGGKR
AtCENP-C  527 ENVNTGSASGLQVENAPEVHKYSHKQT-NKRRKRGSSDSNVKKRSK--------------
HsCENP-C  538 SPVYSNSSV----RNELPMHHNSSR---KSTKKTNQSSKNIRKKT----IPLKRQKTATK
```

```
PpCENP-C  793 RDLDGKTSVYAEGVFPIAGQGVLTNDFLDGKTSTHDEDVLPNAEVEALSSDGHDGGMDYE
AtCENP-C  572 --------------------------------TVHGE-------------TGGD------
HsCENP-C  587 GNQRVQKFLNAEGSGGIVG---------------HDE-ISRCSLSEPLESDEAD------

PpCENP-C  853 NEYDDMSNDTTCIVQKVLSCSINDQEGTSALKECPREIDPLDIDMRQTTS-----TPSRD
AtCENP-C  581 ---------------------------------------------KQMKT-----LPHE-
HsCENP-C  625 -----------LAKKKNLDCS----RSTRSSKNEDNIMTAQNVPLKPQTSGYTCNIPTES

PpCENP-C  908 DIHAGTIAGSVVEYSPAQQDEVHIDQFMNSLQVTPGVDESRLERANTYGESVSNDVNLNG
AtCENP-C  590 ---------------------------------------SRAKK-QTKGKSNEREEK---
HsCENP-C  670 NLDSGEHKTSVLEESGP----------------------SRLN--NNYLMSGKNDV----

PpCENP-C  968 QSPPGNVGRLSPDKELITPGKDGSKRSAEAQDECTPVLRKPKPKRSRKGTKAKKVDSPLD
AtCENP-C  607 ----------KPKKTLTHEGKLFSCRKSLAA-----------------------------
HsCENP-C  702 ------------DDEEVHGSSDDSKQS------------KVIPKNRIHHKLVLPSNTP--

PpCENP-C 1028 AGSKWSDGKRKSTRIRSKPLDWWRGERMLYGRVHSSLNTLIGIKHLSPDPVWPRPRDKKN
AtCENP-C  628 AGTKIEGGVRRSTRIKSRPLEYWRGERFLYGRIHESLTTVIGIKYASPGE--GKRDSRAS
HsCENP-C  736 -------NVRRTKRTRLKPLEYWRGERIDYQGRPSGGFVISGV--LSPDTISSKRKAKEN

PpCENP-C 1088 ----------------------------------------------------KMVPPKFK
AtCENP-C  686 -----------------------------------------------------------K
HsCENP-C  787 IGKVNKKSNKKRICLDNDERKTNLMVNLGIPLGDPLQPTRVKDPETREIILMDLVRPQDT

PpCENP-C 1096 VDSFVSDEHKEL---------------LRLAAQ---------------------------
AtCENP-C  687 VKSFVSDEYKKL-------------------------------------VDFA----ALH
HsCENP-C  847 YQFFV--KHGELKVYKTLDTPFFSTGKLILGPQEEKGKQHVGQDILVFYVNFGDLLCTLH

PpCENP-C      ---------------------------------------
AtCENP-C  706 ---------------------------------------
HsCENP-C  905 ETPYILSTGDSFYVPSGNYYNIKNLRNEESVLLFTQIKR
```

```
PpCENP-O-1    1 ------------------------------------------------------------
PpCENP-O-2    1 MAEEKEKRKVGERRSAQRIKRVLADTTPKHLEQLTKQARMLAGDDDGEADFQDSSMDVIR
AtCENP-O      1 ---------MGE---------------------------MIVSMD------QDIRLDTTR
HsCENP-O      1 MEQANPLRPDGESKGG-----------------------VLAHLE---------------

PpCENP-O-1    1 ------------------------------------------------------------
PpCENP-O-2   61 RRLGHLQQRHTVLREKLTNANPPQRSL------VARLQEEFE------NEPEGKCLEAEE
AtCENP-O     19 ARLSNLLKRHRELSDRLTRDS--DKTM------LDRLNKEFEAA--RRSQSQEVFLDGEE
HsCENP-O     23 -RLETQVSRSRKQSEELQSVQAQEGALGTKIHKLRRLRDELRAVVRHRRASVKACIANVE

PpCENP-O-1    1 --------------------------------------------------------MKGG
PpCENP-O-2  109 WNDSVIKELQKRIYDSDAALSMEDDQEMNEPFEG----KEDAENLTLLVNHKVIRGVKGR
AtCENP-O     69 WNDGLLATLRERVH-------MEADRKADNGNAGFSLVCHPEERITYRVGNKVICCLDGS
HsCENP-O     82 PNQTVEINEQEALEEK-----LENVKAILQAY-----------HFTGLSGK-----LTSR

PpCENP-O-1    5 YVVIRFDTTFENKKFESYYCVLKSNSPMDELHVIEHSIPFFLPVQELEKQHLGKSSKLFF
PpCENP-O-2  165 HIAIRFDTTFEEKKFESYYCVLKSSSPMDKLHVIEHSIPFFLPVRELEKQHLGNSPKLFI
AtCENP-O    122 RIGIQFETSTAGETYEVYHCVLESKSFLEKMIVLEHTIPFFLPLSDLENDLLFSNAKKFI
HsCENP-O    121 GVCVCISTAFEGNLLDSYFVDLVIQKPLR---IHHHSVPVFIPLEEIAAKYLQTNIQHFL

PpCENP-O-1   65 DHMGNVLQAYISRREQVNELKKRKGDLI-GELYHSLSYTLIEIMLK-EPGWQ---VGMSL
PpCENP-O-2  225 DYIGNVLQAYVSRREQVNKLKKRRGELI-GELYHSLSYTLIEIMLK-QPQWQ---VGMSL
AtCENP-O    182 DNVGDLLQAYVDRKEQVRLIKELFGHQI-SEIYHSLPYHMIEFSMD-DCDCK---FVVSL
HsCENP-O    178 FSLCEYLNAYSGRKYQADRLQSDFAALLTGPLQRNPLCNLLSFTYKLDPGGQSFPFCARL

PpCENP-O-1  120 AYHNSDSELPTQSNITVWPLVAHLVSQTAKNSGRGVQKNAAAFRLPINEALFRAMPLPQV
PpCENP-O-2  280 AYHNLDSELPTQSTITTWPLTTDLISQAVIKPGRAVQKNSTAFRLPKAEAFLRTMPLPQG
AtCENP-O    237 RYGDLLCELPTKVRILVWPM-HHLSKKQCTSPGSP----AIPVRLPFAEDAFRIQSLPEA
HsCENP-O    238 LYKDLTATLPTDVTVTC--------------QGVEVLSTSWEEQRASHETLFCTKPLHQV

PpCENP-O-1  180 CDELIIDLRDTLGLS-PLEQAIEAPS------------ASTQSPMEIEI-----EAPMEF
PpCENP-O-2  340 CEELLADLRDTLGLSPPPEQATEAPPPPTVALIEAEMVAPTKPVMQIQMDDLVEDPPMDM
AtCENP-O    292 YAEIMPNM--------P-------------------------------------NEIRQL
HsCENP-O    284 FASFTRK-----------------------------------------------GEKLDM

PpCENP-O-1  222 SQEAPH
PpCENP-O-2  400 PQVTPR
AtCENP-O    307 FQTSPS
HsCENP-O    297 SLVS--
```

```
PpCENP-S     1 ------------------------MESG--------------------------------
AtCENP-S     1 MFNISYAKRNAKYLFKLLAWCMMIEEAGPNIGNKQKVVLQLSKMLIMYIILHINAAFWTW
HsCENP-S     1 ------------------------------------------------------------
PpTaf9       1 ------------------------------------------------------------
AtTaf9       1 ------------------------------------------------------------
HsTAf9       1 ------------------------MESG--------------------------------

PpCENP-S     5 ----------------------------------------AGGERTRDSDGD-----CDM
AtCENP-S    61 RGYLKRCVLHFVFPRIFLPLLSDSPTITQAKKPSYCFAMDVGGEDISDLQVD-----QIV
HsCENP-S     1 -----------------------------------------------------------M
PpTaf9       1 ----------------------------------------MA--EEDDMPRDAKTVKTIL
AtTaf9       1 ----------------------------------------MAGEGEEDVPRDAKIVKSLL
HsTAf9       5 ---------------------------------------KTA--SPKSMPKDAQMMAQIL

PpCENP-S    20 EEYGQL-QQQLHSSKMRLHDRLQVATIATAERTAHKCGMKVSSRVMSALTDLTFKFAERL
AtCENP-S   116 EEY---------SMDDLIRDRFRLSAISIAEAEAKKNGMEIGGPVVACVADLAFKYAENV
HsCENP-S     2 EEEAETEEQQRFSYQQRLKAAVHYTVGCLCEEVALDKEMQFSKQTIAAISELTFRQCENF
PpTaf9      19 ESMGVT---------------------------------RFEPRVINQFLDLWYRYVVDV
AtTaf9      21 KSMGVE---------------------------------DYEPRVIHQFLELWYRYVVEV
HsTAf9      24 KDMGIT---------------------------------EYEPRVINQMLEFAFRYVTTI

PpCENP-S    79 AVDAELFAQHAGRKKVDTDDVLLVARRN----------EDVLASLRLLAQSFA-------
AtCENP-S   167 AKDLELFAHHAGRKVVNMDDVVLSAHRN----------DNLAASLRSLCNEL--------
HsCENP-S    62 AKDLEMFARHAKRTTINTEDVKLLARRS----------NSLLKYITDKSEEIAQ------
PpTaf9      46 LGDAQTYAEHAGKAAIDCDDVKLAIQSRVNSSFQQPPPRELLVDLAKARNSIPLPKIIGT
AtTaf9      48 LTDAQVYSEHASKPNIDCDDVKLAIQSKVNFSFSQPPPREVLLELAASRNKIPLPKSIAG
HsTAf9      51 LDDAKIYSSHAKKATVDADDVRLAIQCRADQSFTSPPPRDFLLDIARQRNQTPLPLIKPY

PpCENP-S   122 ------------------------KSK---------------------------------
AtCENP-S   209 ------------------------KAK---------------------------------
HsCENP-S   106 --------------INLERKAQKKKKS---------------------------------
PpTaf9     106 HGISLPPEADTLIYPNYQL----------------------------NVPTRTTFRDMDD
AtTaf9     108 PGVPLPPEQDTLLSPNYQL----------------------------VIPKKSVSTEPE-
HsTAf9     111 SGPRLPPDRYCLTAPNYRLKSLQKKASTSAGRITVPRLSVGSVTSRPSTPTLGTPTPQTM

PpCENP-S   125 -----------------EKGPEKKRKKAV-ADVP--------------------------
AtCENP-S   212 -----------------EPQSERKRKKGS-AKKE--------------------------
HsCENP-S   119 -----------------EDGSKNSRQPAE-AGVV--------------------------
PpTaf9     138 DIEWEDDDDKEDGGK--EVVQPTHLQ----------------------------------
AtTaf9     139 --ETEDDEEMTDPGQ--SSQEQQQQQQQT-SDLP--------------------------
HsTAf9     171 SVSTKVGTPMSLTGQRFTVQMPTSQSPAVKASIPATSAVQNVLINPSLIGSKNILITTNM

PpCENP-S   141 ----DT---------------------------------
AtCENP-S   228 ----DKASSSNAVRITTDL--------------------
HsCENP-S   135 ----ESEN-------------------------------
PpTaf9     162 ----DSGR-----KVSFSITGKDSKP-------------
AtTaf9     168 ---SQTPQ-----RVSFPLSRRPK---------------
HsTAf9     231 MSSQNTAN-----ESSNALKRKREDDDDDDDDDDDYDNL
```

```
PpCENP-X    1 ME--HLTSTFQPE--------IFKFVWAQTAKKRSKEDSDEADPEVAAAGAGPSKKSRGT
AtCENP-X    1 MD---ANNTFDSDLI----HAIFKHIWAR--RFRERERSDAIDATEAEVALGTTKKNRLA
HsCENP-X    1 MEGAGAGSGFRKELVSRLLHLHF--------------------------------KDDKT

PpCENP-X   51 NINSDALKLSCEYLRLFVTEAVERAALVAEAEGCTTIEGTHFERILPQLLLDF
AtCENP-X   52 SANANALKLSCELLKSFVSEAVQRAAIIAEAEGMEKIEATHLERILPQLLLDF
HsCENP-X   29 KVSGDALQLMVELLKVFVVEAAVRGVRQAQAEDALRVDVDQLEKVLPQLLLDF
```

```
PpKNL1    1 -----------------------MDVREAPPTPTLDFRELDETTTFELYDGSSAVQNRKR
AtKNL1    1 ------------------------MASEKPEDP------MNNTAGIG-TDEESIAQRRKR
HsKNL1    1 MDGVSSEANEENDNIERPVRRRHSSILKPPRSPLQDLRGGNER-----VQESNALRNKKN

PpKNL1   38 RRSSLRRVSFA--EQPSIHVFARDDDYETPPEGTTSHLSAQSTPTSVSRSPQRRSRR--L
AtKNL1   30 ----LRRVSFADREITSVHIFNRDEDYETPPN------------TSAAK-PQ--------
HsKNL1   56 ----SRRVSFAD----TIKVFQTESHMKIVRKSEMEGCSAM-VPSQLQLLPPGFKRFSCL

PpKNL1   94 AARGGEQNENEIDKENVPISVGSNARQGRRGRRPLALRDTQKEKE----EDFLKPSNWLD
AtKNL1   65 --NGGDTSEPDEDNKVIRFFGELSDREDTDG-------DGDGEYEPILDKSFLRP-KYSP
HsKNL1  107 SLPETETGEN------------------------LLLIQNKKLED-----------NYCE

PpKNL1  150 DHENSAFGSCDDNTGPVDL----RHISF-------ENSTVALEEDD--------------
AtKNL1  115 SSGGSTVGSATSDNGTLQLLCEFRVLFFEF---LAESILFSLPEDNFFGPVSSH--FINP
HsKNL1  132 ITGMNTLLSAPIHTQMQQK--EFSIIEHTRERKHANDQTVIFSDENQMDLTSSHTVMITK

PpKNL1  185 -------------NMTMDSQTF------HTR-MSQ-------------------------
AtKNL1  170 GRLLDTPISEEHHEMTMDSTAFS----MHFRSLAR-------------------------
HsKNL1  190 G-LLDNPISEK--STKIDTTSFLANLKLHTE-DSRMKKEVNFSVDQNTSSENKIDFNDFI

PpKNL1  200 -----------------------VFAKQPLNRLSEEK----LVEVTTPGQLT-----DME
AtKNL1  201 -----------------------SESGDVRTPTSSHL----LVEEKTPTEVTSR--SDTG
HsKNL1  246 KRLKTGKCSAFPDVPDKENFEIPIYSKEPNSASSTHQMHVSLKEDENNSNIT-RLFREKD

PpKNL1  228 LTTALTQRQALDRERRVLEPPTWHRGSGDSDMSQGSLNEISIF-------------RVL-
AtKNL1  232 SAMVLTEPKKLFPK----SPVPVDKGSGGRDS-----NDMSI------------------
HsKNL1  305 DGMNFTQCHTANIQ--TLIPTSSETNSRESKG-----NDITIYGNDFMDLTFNHTLQILP

PpKNL1  274 --------------------------SKKRSTAKESDSHDY-------------------
AtKNL1  265 -------------------------------VGENSRRYDYGYLS--------PTLAALM
HsKNL1  358 ATGNFSEIENQTQNAMDVTTGYGTKASGNKTVFKSKQNTAFQDLSINSADKIHITRSHIM

PpKNL1  289 ----------------------E-----------------------------ENTTDVAN
AtKNL1  286 GDESK-------------ELLPE-----------------------------DNTVE-AR
HsKNL1  418 GAETHIVSQTCNQDARILAMTPESIYSNPSIQGCKTVFYSSCNDAMEMTKCLSNMRE-EK

PpKNL1  298 NWIEDDAQS--------------------RGSMSSMQMKK--------------------
AtKNL1  303 SPIDDFSSSLPNGCIPIGLQESGSQRYTKEASLSSSTIRRQ-------------------
HsKNL1  477 NLLKHDSNY--------------AKMYCNPDAMSSLTEKTIYSGEENMDITKSHTVAIDN

PpKNL1  318 ------------------------IGLMRDMDD-VVSMETSS-PQSNRER----------
AtKNL1  344 --------------------SAFLVGMLPQ------SLSCVT-PSPTQGG----------
HsKNL1  523 QIFKQDQSNVQIAAAPTPEKEMMLQNLMTTSEDGKMNVNCNSVPHVSKERIQQSLSNPLS

PpKNL1  342 -SENLRDEHVMAYEGEHAPDTRSSRDSDG---------------SEGSHLQGSRTRERVD
AtKNL1  367 -SFMSRETRALV---ESLSTIQKSKSRLGL-IP----------PSPGSALSQRIEKSKLQ
HsKNL1  583 ISLTDRKTELLS--GENMDLTESHTSNLGSQVPLAAYNLAPESTSESHSQSKSSSDECEE

PpKNL1  386 LNGRR------------------------------------------PSTLLQNRSSRFD
AtKNL1  412 LSGHRFLTTPSIGREEI---------------GVLR-----DKHADIPITNLEALLSKHD
HsKNL1  641 ITKSR---NEPFQRSDIIAKNSLTDTWNKDKDWVLKILPYLDKDSPQSADCNQEIATSHN

PpKNL1  404 -------------NDDTVSMETT--------------SQSHADQNDVTMEIDS--GTDER
AtKNL1  452 -------------NRTPISEEKS--------------MPDKCISGALSHAVDT--SDDNR
HsKNL1  698 IVYCGGVLDKQITNRNTVSWEQSLFSTTKPLFSSGQFSMKNHDTAISSHTVKSVLGQNSK

PpKNL1  435 FSIPSKHS---------------SDTLEGSVKTASEHTALD---PSAHQ-----------
AtKNL1  483 TPVPEEKGI----PDQC-----ISGALSHAVDTSDDNKTPV---P---------------
HsKNL1  758 LAEPLRKSLSNPTPDYCHDKMIICSEEEQNMDLTKSHTVVIGFGPSELQELGKTNLEHTT

PpKNL1  466 ----SSEHNITLESDRIALKPPVHSG---------ASDDSSVLLKHYEQRLKGLSASTSE
AtKNL1  516 -----EEKGIPDQCSSGALNPAVDT-----------SDDNRTPV----QEKKGLPDQCSS
HsKNL1  818 GQLTTMNRQIAVKVEKCGKSPIEKSGVLKSNCIMDVLEDESVQKPKFPKEKQNVKIWGRK

PpKNL1  513 KEISVHVD-----SKMDSQSQSLKSQ----------------------GYAPSM------
AtKNL1  556 GALSPAVD-----TSDDRPPVSEKK---------------------------GI------
```

```
HsKNL1  878 SVGGPKIDKTIVFSEDDKNDMDITKSYTIEINHRPLLEKRDCHLVPLAGTSETILYTCRQ

PpKNL1  540 --------------------PEERLAR-------LQVDRHA-----QALERQELFSSHRAAN
AtKNL1  578 --------------------P----------------DQHSCGALIPAVDISDVFAR-----
HsKNL1  938 DDMEITRSHTTALECKTVSPDEITTRPMDKTVVFVDNHV---ELEMTESHTVFIDYQE--

PpKNL1  570 KESGKSVNLQARDGFVNHGIEQ-------GPLLSLPDENT---------------------
AtKNL1  599 ---------RSPEGNTNSEIE--------GSLLCKQQQRN---------------------
HsKNL1  993 ---------KERTDRPNFELSQRKSLGTPTVICTPTEESVFFPGNGESDRLVANDSQLTP

PpKNL1  603 --------------ESMDITKTWNGRVPQHNSVPTRSSD---SAKS---YFSLDKDQ---
AtKNL1  622 --------------QAASTPE--------KFVSSPTNLSNATTSASE---NFVPLQDQ---
HsKNL1 1044 LEEWSNNRGPVEVADNMELSKSATCKNIKDVQSPGFLNEPLSSKSQRRKSLKLKNDKTIV

PpKNL1  640 --RMDMSDV---------DITDKYAEGI--TEAIPKWKD--VLGA------YNCDWPSVE--
AtKNL1  655 --EQHSKDIEKSETGDGNVTKEYAS---------------------------NCS-------
HsKNL1 1104 FSENHKNDM---------DITQSCMVEIDNESALEDKEDFHLAGASKTILYSCGQDDMEIT

PpKNL1  679 ---------------------PLNRIL----------------------QKKHDVNAEDSPV
AtKNL1  681 ----------------------MNTLS----------------------EKVDSLLAESSVL
HsKNL1 1156 RSHTTALECKTLLPNEIAIRPMDKTVLFTDNYSDLEVTDSHTVFIDCQATEKILEENPKF

PpKNL1  698 CVRKA---GVDFDKALPAKRDAG-LSKATEDR----------------------------
AtKNL1  699 LT-------------------DTGFLNGSAQQR----------------------------
HsKNL1 1216 GIGKGKNLGVSFPK------DNSCVQEIAEKQALAVGNKIVLHTEQKQQLFAATNRTTNE

PpKNL1  726 ----------ETVLSRDLDES-TVE------FHLNMGKKRNI-FQN---------PEDTFS
AtKNL1  713 ----------EKDSVRN---------------------KKQN------------------
HsKNL1 1270 IIKFHSAAMDEKVIGKVVDQACTLEKAQVESCQLNNRDRRNVDFTSSHATAVCGSSDNYS

PpKNL1  760 F---------------------KGHQKS----------IKETSVSPPLMLNEIPKPMF----
AtKNL1  724 ----------------------------------------RTNISAAHILLKDNNPF----
HsKNL1 1330 CLPNVISCTDNLEGSAMLLCDKDEEKANYCPVQNDLAYANDFASEYYLESEGQPLSAPCP

PpKNL1  787 ---------------------------------SNVREKVALIEANQRSQIA---------
AtKNL1  741 ----------------------------------KVHCETEVISAEDFTAVAKEN------
HsKNL1 1390 LLEKEEVIQTSTKGQLDCVITLHKDQDLIKDPRNLLANQTLVYSQDLGEMTKLNSKRVSF

PpKNL1  806 --PNGSERDFQDEDTTGDFTGQHNW----------RAEVSSDM------------------
AtKNL1  762 -LPSTS------------------------------GSSSVDR------------------
HsKNL1 1450 KLPKDQMKVYVDDIYV---IPQPHFSTDQPPLPKKGQSSINKEEVILSKAGNKSLNIIEN

PpKNL1  837 STTPLS-PRRPDYF------------QRLITKPQVAPIASETGLADSPSFVDGITAAI---
AtKNL1  774 SKNEASHAKGPSRL------------KRKAEDVDCAARNCSPKVERSTKYISNSVMEH---
HsKNL1 1507 SSAPIC-ENKPKILNSEEWFAAACKKELKENIQTTNYNTALDFHSNSDVTKQVIQTHVNA

PpKNL1  882 -------------------PNVQDL------------------------------------
AtKNL1  820 -------------------PD-GNI------------------------------------
HsKNL1 1566 GEAPDPVITSNVPCFHSIKPNLNNLNGKTGEFLAFQTVHLPPLPEQLLELGNKAHNDMHI

PpKNL1  888 --------------LAADAGTPESELGWN--------PRKIVW------------------
AtKNL1  825 --------------DANDCRRVREQVNWVE------IPGKV--------------------
HsKNL1 1626 VQATEIHNINIISSNAKDSRDEENKKSHNGAETTSLPPKTVFKDKVRRCSLGIFLPRLPN

PpKNL1  909 -----------------------------------------------SAQQSNRQTME--
AtKNL1  846 ------------------------------------------------SKEINQMLAP--
HsKNL1 1686 KRNCSVTGIDDLEQIPADTTDINHLETQPVSSKDSGIGSVAGKLNLSPSQYINEENLPVY

PpKNL1  920 -----DRDDLR-------------QPRTVQMEDDTMDSLPDQLRT---------PKGLQERD
AtKNL1  856 -----LADKLN-------------SRLICKLEDILTH--------------------MKKV
HsKNL1 1746 PDEINSSDSINIETEEKALIETYQKEISPYENKMGKTCNSQKRTWVQEEEDIHK--EKKI

PpKNL1  955 HKFE---CMTEEAVKTLNQVSHSKEVRNSVASKMLMRLMS---------IQDKGRAEAESS
AtKNL1  879 HLCEM-LCLQIQSQKVCDHLSGAKTKRRVESRSLLCKLA----------YDKAKLELLH-
HsKNL1 1804 RKNEIKFKFSDTTQDREIFDH--HTEEDIDKSANSVLIKNLSRTPSSCSSSLDSIKADGTS-
```

```
PpKNL1 1004 SDGESQQILRRTLQFHPESDSQLPSQSLR----EEDTGHQMSAG----------------
AtKNL1  927 ---LKKEIMMK--KFQAVSTGVQTSETLR----LNCANFLRQHGF---------------
HsKNL1 1861 ------------LDFSTYRSSQMESQFLRDTICEESLREKLQDGRITIREFFILLQVHIL

PpKNL1 1044 -------------------DPDDLM--------KV---------------ESIKDMDEET
AtKNL1  963 ----RSTGL---------LNPDQAQ--------EVII-----TGKRAEITQEIKEID---
HsKNL1 1909 IQKPRQSNLPGNFTVNTPPTPEDLMLSQYVYRPKIQIYREDCEARRQKI-EELKLSASNQ

PpKNL1 1062 DSI---------ETKTHSSRQE--------SRTKSLLQELQLSEEKLGALKRRSEELRMR
AtKNL1  994 ------------------------------SKIKNLIQCFTACDTMTGPQPAYADTI-MI
HsKNL1 1968 DKLLVDINKNLWEKMRHCSDKELKAFGIYLNKIKSCFTKMTKVFTHQGKVALYGKLV-QS

PpKNL1 1105 AQDCHQAIAKKCRD--------DSCF------------------------EM----KELA
AtKNL1 1023 AEE---------------------------------------------------------
HsKNL1 2027 AQNEREKLQIKIDEMDKILKKIDNCLTEMETETKNLEDEEKNNPVEEWDSEMRAAEKELE

PpKNL1 1129 AFCSQ-------------------AHVSKLKKRC----QLLNC------VQWWNVKS-AK
AtKNL1 1026 ----------------------------TLKKR-------MSCRSLRQDILIWKVDSLGE
HsKNL1 2087 QLKTEEEELQRNLLELEVQKEQTLAQIDFMQKQRNRTEELLDQLSLSE----WDV---VE

PpKNL1 1159 TGDGQVLHEFKYWSLLTHSIFERSAINLES------FVS---RSLIDLNDEKIDQ-----
AtKNL1 1051 WNDCQSI-------VLNYSGVFNQRLTLKPGHPSCVLVS---NSLSD----TFVK-----
HsKNL1 2140 WSDDQAVFTFVY-DTIQLTITFEESVVGFP------FLDKRYRKIVDVNFQSLLDEDQAP

PpKNL1 1205 ---------VYSHMNASLAW---------RWFLSG---LDEIIQSQSLFYHVQCINSKVM
AtKNL1 1092 ---------HFPEMNVSIAFNSMFNAEDSRRYIGGSNTLLEITQKTSLLLHN--------
HsKNL1 2193 PSSLLVHKLIFQYVEEKESW---------KKTCTTQHQLPKMLEEFSLVVHH-CR-----

PpKNL1 1244 ILLDLMEEVKSCR---KYLVIPYFRLQTDTNLQLGLKFSNSELLFDFVVLLNIRQVVQMR
AtKNL1 1135 -LLDVAEEFHLAQMNIPNLVQGNFDSPSAEQLHLQISFLDCTNLRKLSVILDVTCLIHGK
HsKNL1 2238 -LL--GEEIEYLKRWGPNYNLMNIDINNNE---LRLLFSSSAAFAKFEITL----FLSAY

PpKNL1 1301 YSRRYELDRAVEMSQKLDCDVEMESEGRFFGKKVSAV-------EIKSCVSSVVSGHQLL
AtKNL1 1194 YP-----------SDVVPCEFRKVS-----GTKRDGVVSKQLKKEIESTIDDVGVGYPRI
HsKNL1 2288 YP-------SVPLPSTIQNHVGNTSQDDI-ATILSKV--PLENNYLKNVVKQI---YQDL

PpKNL1 1354 SRVCACVNTLIEKIKGESNYR
AtKNL1 1238 LRLCRCISKALQSEKR-----
HsKNL1 2335 FQDCHFYH-------------
```

```
PpMis12    1 MEVDTRDKILAMYPVHFLNFDKDA-----------FTADVVNAVIEISMSNFTEMERCAT
AtMis12    1 MEGSKSEAVFDS-----MNLNPQI-----------FINEAINSVEDYVDQAFDFYARDAS
HsMis12    1 MSVDPM-----TYEAQFFGFTPQTCMLRIYIAFQDYLFEVMQAVEQVILKKLDGIPDCDI

PpMis12   50 RMLNITSTESQ--EALQQGLSAVFEKFLSKFIEEDLAPWEAYCREVCFKVPDGMVLPEKL
AtMis12   45 KSLKIKGSDKQKSQALSNGIARVRGLLLS-VIDNRLKLWESYSLRFCFAVPDGFVLPKSE
HsMis12   56 SPVQIRKCTEK------------FLCFMKGHFDNLFSKMEQLFLQLILRIPSNILLPEDK

PpMis12  108 DASVVAMEDA-DAK-LDAELISLRERKAT--AEKEAAELRRDVKALE----------ALV
AtMis12  104 ESSSVHQDGLYDLE-LDAELDSLRDKLNV--VGKRSVELDSELQALERSSVSRERSLRLV
HsMis12  104 CKETPYSEE--DFQHLQKEIEQLQEKYKTELCTKQAL-----LAELEEQ--------KIV

PpMis12  154 EAKA-NFMDAFDQLQN---LPLVDDLGNVVRELRKNV-----------EEANALRAQRYQ
AtMis12  161 NEA----LELYDESSM---DEIFKEMTKMASELRASVERLKTRRMKASESAKVKRLKNHG
HsMis12  149 QAKLKQTLTFFDELHNVGRDHGTSDFRESLVSLVQNSRKLQNIRDNVEKESKRLKIS---

PpMis12  199 RLFPADTSDAL--------------
AtMis12  214 KEFSAMTFDGKLEDLEKFQAELRKM
HsMis12      -------------------------
```

```
PpNnf1    1 ---------MGNDGEKK---------------LRYEDLWETLNIASRGALVAVSENEFEK
AtNnf1    1 ---------MEKPGHETDIAG-----------SRRTHLKKSFKSTLRHLLTACSKQDFVD
HsNnf1    1 MAEASSANLGSGCEEKRHEGSSSESVPPGTTISRVKLLDTMVDTFLQKLVAAGSYQRFTD

PpNnf1   37 CFPEFTNEQKEKLFLIYTRVVAKLQEDVMLEFKPLCEAWELKEVLEELEELIE-------
AtNnf1   41 IFSKFSGAEQELLFQLYTRVVVNLHQTIEEEFDEQCHETQVGPILDTVEELVE-------
HsNnf1   61 CYKCFYQLQPAMTQQIYDKFIAQLQTSIREEISDIKEEGNLEAVLNALDKIVEEGKVRKE

PpNnf1   90 ----------RHELGDVLT-----------------SRSAPLTDAVRAMTTKIKQEEVDK
AtNnf1   94 ----------EQSLDPLFS-----------------DKTDVMAIAYDLTTS--KKNEIQK
HsNnf1  121 PAWRPSGIPEKDLHSVMAPYFLQQRDTLRRHVQKQEAENQQLADAVLA-----GRRQVEE

PpNnf1  123 LKLLVQQEEEHVAQLRLQREALQAKAREEPTALAATLGKVRRERVELSL-----------
AtNnf1  125 LTVLLQRAEEQ----NRQKEARISMLKKQTQDLSGTADRIEKLNAGVSGYFEGKDKLPPI
HsNnf1  176 LQLQVQAQQQAWQALHREQRELVAVLREPE------------------------------
```

```
PpDsn1-1    1 ----MAAL------EFEAENDDGFVFKVRKRR----HLEYTSVSPLD------TMEPLKAL
PpDsn1-2    1 ----MAAS------EFETVNDDGFVFKVRKRR----RLEEINVKPL--------------A
AtDsn1      1 MDSPMSTSSIDGDEDWECYEDDGFVYNRKKRTRFADAEETSKPPD---------PELDRV
HsDsn1      1 ----MTSVT-----RSEIIDEKGPVMSKTHD----HQLESSLSPVEVFAKTSASLEMNQG

PpDsn1-1   41 VKERHSHEDPEPVAGHPCDLEKKAKRLLT---------LKEMYEAELEAWKKL--------
PpDsn1-2   33 VKEGQRHEDQEPVEEQSVKLEKKAKRLMT---------LKAMYEAELEAWKKL--------
AtDsn1     52 VEERNRR-------------IRKKRLLVK---------LKRKYQSEIDQWEILS-------
HsDsn1     48 VSEERIHLGSSPKKGGNCDLSHQERLQSKSLHLSPQEQSASYQDRRQSWRRASMKETNRR

PpDsn1-1   85 ------------------------------------------------------------
PpDsn1-2   77 ------------------------------------------------------------
AtDsn1     84 -----------------------------------------------------NSFNA-
HsDsn1    108 KSLHPIHQGITELSRSISVDLAESKRLGCLLLSSFQFSIQKLEPFLRDTKGFSLESFRAK

PpDsn1-1   85 ------------------------------------------------------------
PpDsn1-2   77 ------------------------------------------------------------
AtDsn1     89 -------------------------------------------MQEKADRF---------
HsDsn1    168 ASSLSEELKHFADGLETDGTLQKCFEDSNGKASDFSLEASVAEMKEYITKFSLERQTWDQ

PpDsn1-1   85 -----------------EAAAVPLVFEEPLSNSDTP-----------LSSPEQ-------
PpDsn1-2   77 -----------------EEAAAPPVPDEP-SSEKSP------------SSPEH-------
AtDsn1     97 ------QTAQR-----EERLNANETMSFPGNSSSTTEGGREFGGEDASKSP---------
HsDsn1    228 LLLHYQQEAKEILSRGSTEAKITEVKVEPMTYLGSSQ------NEVLNTKPDYQKILQNQ

PpDsn1-1  110 ----DPHAEFLKELKIEVENMEEYIKRLQDHIVLAESTYKLRVKEMREDANFRPADQIVL
PpDsn1-2  100 ----DLHAEFLKELKIEVENVEEYIKRLQDHIVIAESTYKLCVKEMREDANFRPADEIVL
AtDsn1    137 -------SSMLDQLLFTVERQEAVINEVSK---LCEVTENICRVEEEE------------
HsDsn1    282 SKVFDCMELVMDELQGSVKQLQAFMDESTQ--CFQKVSVQLGKRSMQQ-LDPSPARKL-L

PpDsn1-1  166 ALTDQATSDVDVLATQGSLSSSASLNDL
PpDsn1-2  156 ALTGQAISD-DVIATQASLGSSVSLSDV
AtDsn1    175 --TKQSFFDLPIWSSPTDLMASLCGD--
HsDsn1    338 KLQLQ---------NPPAIHGSGSGSCQ
```

```
PpNUF2     1 -MQFSFPVLERKEILSCLS------ADAGI-TLEEQHLV-KPSMETVWPVYESLVLNIMG
AtNUF2     1 MSAYEYPRLSRSDIITALK-------DAQIASVTETDLK-TPTSDFVSELYTRILIYLDA
HsNUF2     1 METLSFPRYNVAEIVIHIRNKILTGADGK--NLTKNDLYPNPKPEVLHMIYMRALQIVYG

PpNUF2    52 ITREELQQPAFHAI---EKLEYPELHEESVGILASIRAISKLMAVTGVDD----FSTMDI
AtNUF2    53 LDEEEKGQVDFEAL---EQLENPDHHATS---MQAMKLYCKVKDMLEMLDCPLPISFKDL
HsNUF2    59 IRLEH-----FYMMPVNSEVMYPHLMEGFLPFSNLVTHLDSFLPICRVND----FETADI

PpNUF2   105 FKPESKRTITHLSAIINLLKFKQGKE---RDLAE---VLDERERAFVLYQQSEERLAALK
AtNUF2   107 LRPESSRTEFFISALLNYGLYKDSKMDLIRPKAEELGLLDEQRK-------QCEAKVAQLN
HsNUF2   110 LCPKAKRTSRFLSGIINFIHFREACR---ETYME---FLWQ-------YKSSADKMQQLN

PpNUF2   159 NEI----EAL-EADRLAQQPAVQALEAETKVLNQEIQALNKQHGALQSDIRA----LKQE
AtNUF2   161 AEIGEFDEAV-ERD----LPFVQELEANIEQLNKKILELNNQQMSLRATFQK----MREK
HsNUF2   157 AAH---QEALMKLERLDSVP-VEEQE-EFKQLSDGIQEL---QQSLNQDFHQKTIVLQEG

PpNUF2   210 GQAVAEEISAE-------KYSLIQQKEEQERLKSQIVQSPQKLQRSLAEKKAALISTKAS
AtNUF2   212 STQMDNEISKA-------EFDLVETVQENANLRSQIVQSPDKLQGALEEKKLVLGETKKA
HsNUF2   209 NSQKKSNISEKTKRLNELKLSVVSLKEIQESLKTKIVDSPEKLKNYKEKMKDTVQKLKNA

PpNUF2   263 AEEAQQLLN----------SFKGKLEAYNKVEKKL----KKLFGMLDEVEKLWVKQKATI
AtNUF2   265 EQSAMVTFQ----------EKAAILEVFEKALKKI----LKSSSQLQLINEQVTNAKTVE
HsNUF2   269 RQEVVEKYEIYGDSVDCLPSCQLEVQLYQKKIQDLSDNREKLASILKESLNLEDQIESDE

PpNUF2   309 KEKKAFK------------AQVKSIEEE--IESLDTELSQLKFQE----QHWLELTEKLE
AtNUF2   311 KEFKALKDKLSEDGVAYKSLEAKVVERERIVEQLNESLKQLEKEKAVMFDDWTKQLNELK
HsNUF2   329 SELKKLK------------TEENSFKRLMIVKKEKLATAQFKINKK---HEDVKQYKRTV

PpNUF2   351 QQGPKKLQEAKRELEDTEANCLPVLARIEERGAANLKAEAQVKEIREKLEAMTRKKENII
AtNUF2   371 VE----VESRRRELETRQTNVESVVAMVDDN--------TAKTNQVRQSGEAKVKK---LA
HsNUF2   374 IEDCNKVQEKRGAVYERVTTINQEIQKI----------KLGIQQLKDAAEREKLKSQEIF

PpNUF2   411 ARGEAAVQKVMKETLHH---------YRTLLEVGPAALAALPNSRLNRGLQ
AtNUF2   417 AKYEEIVKQ-----FHE---------YTVSFD------AFLPSL-------
HsNUF2   424 LNLKTALEK-----YHDGIEKAAEDSYAKIDE----KTAELKRKMFKMST-
```

```
PpNdc80-1     1 ------------------------------------------------------------
PpNdc80-2     1 MSR-RVSAAVPTRVVGKTARLSNVG------------PAYGIPGI---SNGGKSGLLQRR
AtNdc80       1 -----MRGGAAGK------RRTTVGFGGAPPP-----PP--------PSIEQQRHLFNSR
HsNdc80       1 MKRSSVSSGGAGRLSMQELRSQDVNKQGLYTPQTKEKPTFGKLSINKPTSERKVSLFGKR

PpNdc80-1     1 ------------------------------------------------------------
PpNdc80-2    45 ---ASFHGGK--------SSQPRTDPRPLNDRAFQQECIRSLTGYLLTHGYNVPISTKLL
AtNdc80      37 DSDASFASSRPSSIGL--GGRGASDDR--------SSMIRFINAFLSTHNF--PISIRGN
HsNdc80      61 ---TSGHGSRNSQLGIFSSSEKIKDPRPLNDKAFIQQCIRQLCEFLTENGYAHNVSMKSL

PpNdc80-1     1 -------------FLFQKVNPNLKL-SVEVEEEVPVVFKRLGYPFQISMSALYAAGSPYT
PpNdc80-2    94 VSPASKDVLNIVQFLFQKVDPNLKL-SGKVEEDVPVVFKRLGYPFQISKSALYAAGSPHT
AtNdc80      85 PVPSVKDISETLKFLLSAL--DYPCDSIKWDEDLVFFLKSQKCPFKITKSSLKAPNTPHN
HsNdc80     118 QAPSVKDFLKIFTFLYGFLCPSYELPDTKFEEEVPRIFKDLGYPFALSKSSMYTVGAPHT

PpNdc80-1    47 WPGLLAALVWLIHLL-LHQEATESVVTFE-----------------------TQSYQCFL
PpNdc80-2   153 WPGLLAALVWLIQLL-LHQEATESVETFDDAGA------------RSFLENLSQSYQCFL
AtNdc80     143 WPTVLAVVHWLAELARFHQHLVSNSTSVP---------EDNSM--NFFA---IQSFGHFI
HsNdc80     178 WPHIVAALVWLIDCIKIHTAMKESSPLFDDGQPWGEETEDGIMHNKLFLDYTIKCYESFM

PpNdc80-1    83 AGDDDECERLDCAFRQQFKDCTVETTEKLAKL------KVP-EVEERLRRLSTEASPL--
PpNdc80-2   200 AGDDDECERLDYAFRQQFEDCTAEVMEKVAKL------RVP-EVEEQLQKLSTEVSPLT-
AtNdc80     189 RGEDDKVNDLDSQFLGKLEAEKTSVAETISGC-----EKISGELEAKLESL---------
HsNdc80     238 SGAD------------SFDEMNAELQSKLKDLFNVDAFKLE-SLEAKNRALNEQIARLEQ

PpNdc80-1   134 ----------------------------------WHKQAAEKKLEARKGDLVDKKAEKTV
PpNdc80-2   252 ----------SLESKKADFLSDKQKFNTIISSLQWHKQAAEKKLQERKEDLLAKKAEKNE
AtNdc80     235 --RKGPSKKESLEKVKADLENDVNKFRTIVVEYTDRNPAMEKVVEEKAKELKAKEEERER
HsNdc80     285 EREKEPNRLESLRKLKASLQGDVQKYQAYMSNLESHSAILDQKLNGLNEEIARVELECET

PpNdc80-1   160 IQADIEELHNRIASQEINPADIDRMQKEKDFLDANTRPVVLKDQQLRKAAWDHELASTNK
PpNdc80-2   302 IQADIEELRNRIASQEINLADIERMQKEKDLLDANIQPIVAKDQQLRKAAWEHELATTNK
AtNdc80     293 ISVENKELKKSVELQNFSAADVNRMRRELQAVERDVADAEVARDGWDQKAWELNSQIRNQ
HsNdc80     345 IKQENTRLQNIIDNQKYSVADIERINHERNELQQTINKLTKDLEAEQQKLWNEELKYARG

PpNdc80-1   220 EKDLEILCGEYNERCLRFKPILADGQ------------PLTGSELQITLQIESTVP-KEI
PpNdc80-2   362 EKDLEILCGEHNDRCQRLKPILADGQ------------PLSGAQLQITLQLDRTLP-QEI
AtNdc80     353 FHQIQTLAIDCNQALRRLKLDIQFAVNERG-----------------------ETP-AAV
HsNdc80     405 KEAIETQLAEYHKLARKLKLIPKGAENSKGYDFEIKFNPEAGANCLVKYRAQVYVPLKEL

PpNdc80-1   267 LGTSVA---------------------NEEAISCEQEVYAYKAARQEKLDLLSRLVATIK
PpNdc80-2   409 LGTSVSGGLKQTILEVMESCKRYTNQEREEAITCEKEVYACEAARQEKLDLLSRNVATIK
AtNdc80     389 MGVDYKSVVKPALCSLCDGIKGSSAEKVEELVTLQHHKSEMASKIESKRSLLGSIQLQIN
HsNdc80     465 L------------------------NETEEEIN---KALNKKMGLEDTLEQLNAMITESK

PpNdc80-1   306 KSEALHKASKEKWDLACAVVTNEREELEAKVVAKELAAKRTDKDAEKLTKQ---WEQRYK
PpNdc80-2   469 KLEALYKASKEKWDAAYVAVVNEREELEAKVIAKEVAAKRTDADAEELVKQ---WEQRYE
AtNdc80     449 DLEEKMKLVKKETQELSTKCDLEAKTLVESVKAEALNLEVVEKEAAEFVKA---SELRLQ
HsNdc80     498 RSVRTLKEEVQKLDDLYQQKIKEAEEEDEKC-ASEL---------ESLEKHKHLLESTVN

PpNdc80-1   363 AEVESSSEQVATAKKQWIEVVDFILSAKEQITGLLQVL--KSKTHV---------EAGRL
PpNdc80-2   526 AGTASCADQLASAKKQWIEVVDYILSAKEQITMLLQNL--KTKTHV---------EAGKV
AtNdc80     506 EAVKESEEEVQACAAQLFALIDSISKQKEYMDSKISEI----KTGV--------------
HsNdc80     548 QGLSEAMNELDAVQREYQLVVQTTTEERRKVGNNLQRLLEMVATHVGSVEKHLEEQIAKV

PpNdc80-1   412 KRDLPEF-TAMIALQISWK-KVKIERHASLHI----
PpNdc80-2   575 KRDLSELVSTMAALQV--------------------
AtNdc80     548 ---------ADTASAVSEIYKANFKKNLGI------
HsNdc80     608 DREYEECMSEDLSENIKEI-RDKYEKKATLIKSSEE
```

```
PpSpc24      1 MDYEGEMKEAEEVIGVMKSLSSIVNTDED-------FNTIQQVVQNLESLVNNCLLQQKQ
AtSpc24-1    1 MGNASENFDIEDLMSYGDDLINLLNVKNG-------FDIISQSSEQFKALNFACHEDFNQ
AtSpc24-2    1 MRDQSRNFEM--VISWGDELIHVLDDRKG-------FDVLVQTLEQLRAIPFSCDEDFKE
HsSpc24      1 ---MAAFRDIEEV---SQGLLSLLGANRAEAQQRRLLGRHEQVVERLLETQDGAEKQLRE

PpSpc24     54 VQSCIQGLREKVK-------------AKEQ------RILNAQK---------LNLKDE--
AtSpc24-1   54 IQGSIEDCKTKLYACKKKTEEAYSDIAAED------EIERLQKELDEEMEREFKLKDELR
AtSpc24-2   52 IHESLQDLQKKLDVCKEKTDEANSEIADEE------EIERLQKELDEELELECKLKEELR
HsSpc24     55 ILTMEKEVAQSLLN------------AKEQVHQGGVELQQLEAGLQEAGEEDTRLKASLL

PpSpc24     84 ----ELEELRNNLAEARIEEEKARATAR---------------YAS--------------
AtSpc24-1  108 LVADELKDL--NAQLSSIDEHK-QSTKRKVRDDLRAEKKLS-MYASVTNVIPDID-DPSK
AtSpc24-2  106 FIADELKDL--NSQEALFEEHR-LAIKRNKRDQLRTETKLP-MYASVTRVIPNID-DSLK
HsSpc24    103 YLTRELEEL--KEIEADLERQE-KEVDEDTTVTIPSAVYVAQLYHQVSKIEWDYECEPGM

PpSpc24    111 -----------------------TSVCLLCLPLTHA-----
AtSpc24-1  163 ISGYMVDREKRLIEKFQFETNKMTA-YETCNSIWSIINKQ-
AtSpc24-2  161 TSGYMVGRDKRLMDKFEFDSNKSTSVYETCNGIWDIINRQ-
HsSpc24    160 VKG--IHHGPSVAQPIHLDSTQ-LSRKFISDYLWSLVDTEW
```

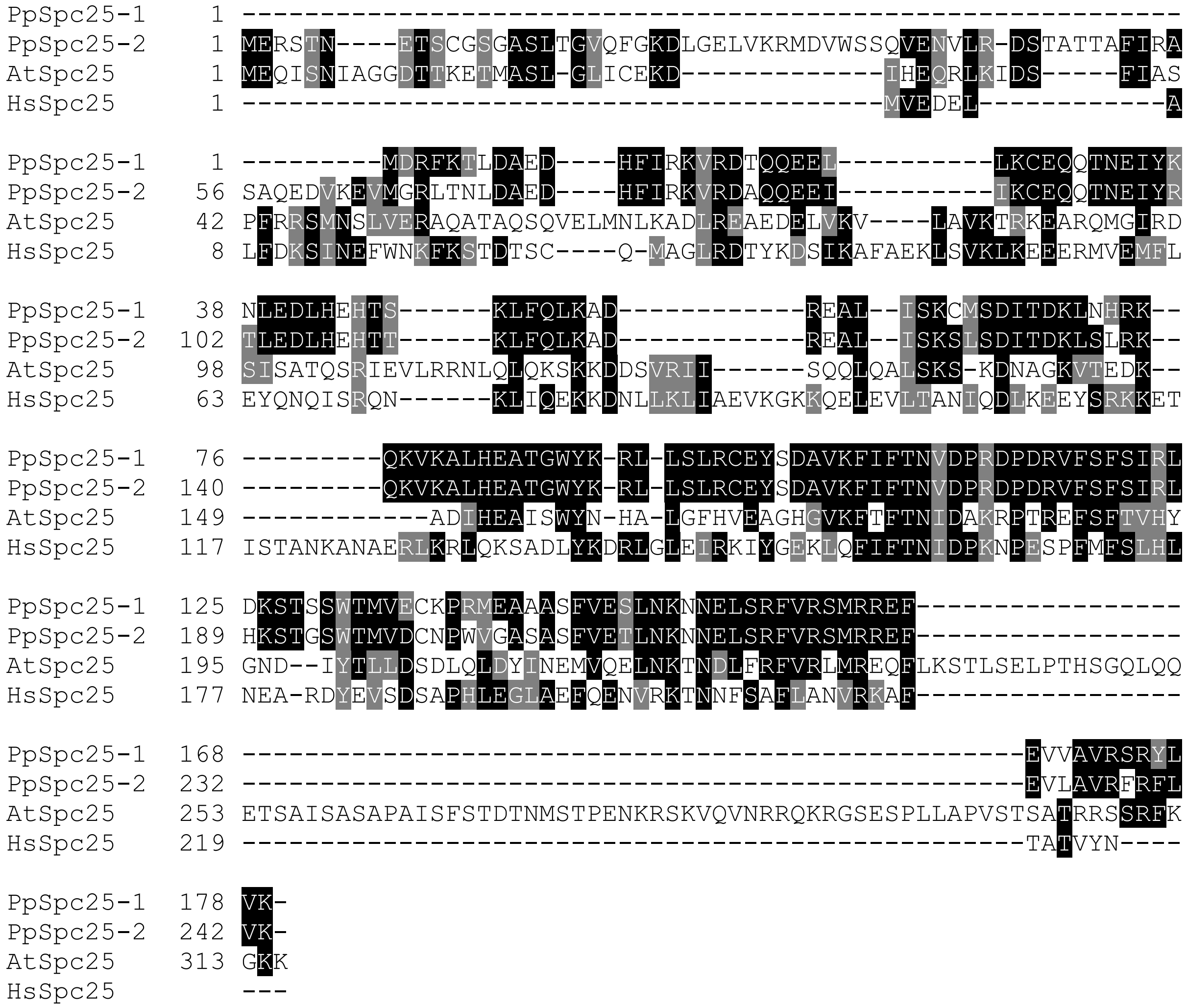
PpSpc25-1 1 ---
PpSpc25-2 1 MERSTN----ETSCGSGASLTGVQFGKDLGELVKRMDVWSSQVENVLR-DSTATTAFIRA
AtSpc25 1 MEQISNIAGGDTTKETMASL-GLICEKD--------------IHEQRLKIDS-----FIAS
HsSpc25 1 --MVEDEL------------A
PpSpc25-1 1 ---------MDRFKTLDAED----HFIRKVRDTQQEEL----------LKCEQQTNEIYK
PpSpc25-2 56 SAQEDVKEVMGRLTNLDAED----HFIRKVRDAQQEEI----------IKCEQQTNEIYR
AtSpc25 42 PFRRSMNSLVERAQATAQSQVELMNLKADLREAEDELVKV----LAVKTRKEARQMGIRD
HsSpc25 8 LFDKSINEFWNKFKSTDTSC----Q-MAGLRDTYKDSIKAFAEKLSVKLKEEERMVEMFL
PpSpc25-1 38 NLEDLHEHTS-------KLFQLKAD------------REAL--ISKCMSDITDKLNHRK--
PpSpc25-2 102 TLEDLHEHTT-------KLFQLKAD------------REAL--ISKSLSDITDKLSLRK--
AtSpc25 98 SISATQSRIEVLRRNLQLQKSKKDDSVRII------SQQLQALSKS-KDNAGKVTEDK--
HsSpc25 63 EYQNQISRQN------KLIQEKKDNLLKLIAEVKGKKQELEVLTANIQDLKEEYSRKKET
PpSpc25-1 76 ---------QKVKALHEATGWYK-RL-LSLRCEYSDAVKFIFTNVDPRDPDRVFSFSIRL
PpSpc25-2 140 ---------QKVKALHEATGWYK-RL-LSLRCEYSDAVKFIFTNVDPRDPDRVFSFSIRL
AtSpc25 149 ------------ADIHEAISWYN-HA-LGFHVEAGHGVKFTFTNIDAKRPTREFSFTVHY
HsSpc25 117 ISTANKANAERLKRLQKSADLYKDRLGLEIRKIYGEKLQFIFTNIDPKNPESPFMFSLHL
PpSpc25-1 125 DKSTSSWTMVECKPRMEAAASFVESLNKNNELSRFVRSMRREF-----------------
PpSpc25-2 189 HKSTGSWTMVDCNPWVGASASFVETLNKNNELSRFVRSMRREF-----------------
AtSpc25 195 GND--IYTLLDSDLQLDYINEMVQELNKTNDLFRFVRLMREQFLKSTLSELPTHSGQLQQ
HsSpc25 177 NEA-RDYEVSDSAPHLEGLAEFQENVRKTNNFSAFLANVRKAF-----------------
PpSpc25-1 168 --EVVAVRSRYL
PpSpc25-2 232 --EVLAVRFRFL
AtSpc25 253 ETSAISASAPAISFSTDTNMSTPENKRSKVQVNRRQKRGSESPLLAPVSTSATRRSSRFK
HsSpc25 219 --TATVYN----
PpSpc25-1 178 VK-
PpSpc25-2 242 VK-
AtSpc25 313 GKK
HsSpc25 ---

```
PpSKA1    1 MEMSATGTAKVDLDALMASFNERINLLRNLTLVRS--GGSHVGELKSLDTALSTAEHHLR
AtSKA1    1 MEGNQAGSS---LDSLIASFNKRIGELQELVIARNMYPASTIPDLSAIDTALSSMELQVQ
HsSKA1    1 -----MASS--DLEQLCSHVNEKIGNIKKTLSLRN------CGQEPTLKTVLNKIGDEII

PpSKA1   59 VIKAFMKR-------EAESLAKVQVLVELSSQQTAKLQQICSNLPARLPGN---------
AtSKA1   58 SIKDRLRE-------ETEAIPKAKKLIEASLKQQGKLQKMSIYAPSHFPDKATMLN----
HsSKA1   48 VINELLNKLELEIQYQEQTNNSLKELCESLEEDYKDIEHLKENVPSHLPQVTVTQSCVKG

PpSKA1  103 ---------------EMHVEPAVASSTSNPQKANNSTAVPAKTAPPKEKRGKEPPPRWYV
AtSKA1  107 SDLNRCLLQENAKQYEQH-------STLSSLKFDEEAAV-----LPKEKKGRGSPPLWYI
HsSKA1  108 SDL----------------------DPEEPIKVEEPEPV---KKPPKEQRSIKEMP--FI

PpSKA1  148 SVDELSSLSSYMRGRLTLEKLNTAIDEMATFATGNAKLLTAPRQKL---GEEGWNRVL--
AtSKA1  155 TVEELNSLSSYMRGRLTLEKVNAAINDMASYAEANAHLIAASKQKL---AENLWEKAL--
HsSKA1  141 TCDEFNGVPSYMKSRLTYNQINDVIKEINKAVISKYKILHQPKKSMNSVTRNLYHRFIDE

PpSKA1  203 ELRDIAIAENVKGKHFFLESDLKG-EVLKLDHTGKAVLTVLRHLGRINEIRCGRNRVFSI
AtSKA1  210 KLRDIVTEQAVKGKHFFLETDMKG-PSLKLDNTGKAILTVLRHLGRISETRIGQNRVIIL
HsSKA1  201 ETKD------TKGRYFIVEADIKEFTTLKADKKFHVLLNILRHCRRLSEVRGGGLTRYVI

PpSKA1  262 NRN-
AtSKA1  269 MKPH
HsSKA1  255 T---
```

```
PpSKA2-1    1 -------------------MEKGFGGMDGAFHVAVKTIDAHFAKADAI---LQIVHSKLE
PpSKA2-2    1 MPILPRRTAYFTGFLCFLRLENFSTG---------KTAHLKFFFSWSLCSWMSIVHSKLE
AtSKA2      1 -------------------MTH------NHHHQAVDNLLNVFSRASHD---LTVVHSKLD
HsSKA2      1 -------------------MEA-----------EVDKLELMFQKAESD---LDYIQYRLE

PpSKA2-1   39 HEFEVTYPGNA---NPLKLLARKKRLQDELPLLREECDKLLAAKQDLIDAARTTLVKNRT
PpSKA2-2   52 HEFEETYPGNA---NPLKLLARTKRLQDELPLLREECDKLLAAKQDLIDTARTTLVKNKT
AtSKA2     33 KEFQQMYPANA---NPMKLIQRIKKLQEDVTLLKHQCLDLLSAKQDLIDKAQTTLVGNCN
HsSKA2     28 YEIKTNHPDSASEKNPVTLLKELSVIKSRYQTLYARFKPVAVEQKESKSRICATVKKTMN

PpSKA2-1   96 LLCQLQARAGLPVSCDADDPIYSSFVKTLEEWDHQLEIVSVTESHEFTRPDLNLELFRSK
PpSKA2-2  109 LLCQLQARAGLLVSSDSEDPVYSSFVKTLEEWDQQMEMVSVTESHQFTRPDLNLELFRSK
AtSKA2     90 LIQKMNASLGESTNGDTDDAL-ADFNQIIDEWTMQVRSRTVGETEDADKEDINKMLFSAI
HsSKA2     88 MIQKLQKQ--------TDLEL-SPLTK---------EEKTAAEQFKFHMPDL--------

PpSKA2-1  156 VFKRDE
PpSKA2-2  169 VFKRDE
AtSKA2    149 CHTN--
HsSKA2        ------
```

```
PpSKA3-1    1 MFSGKRLDTVSEVSPMASQRLPCTSMLATAYCNRLDQFCAQLQNNCSSLLSIAKVKPKPE
PpSKA3-2    1 ---------------MASQRAPSTSMLAAAWCDRLNQFCTQLQKNCSSLHSIVQRKPESQ
AtSKA3      1 ----------------MEEELKSLS-------KSLGGFCNHLQSSCDAFNHSLQRRPIP-
HsSKA3      1 -------------------------------MDPIRSFCGKLRSLASTLDCETARLQRA-

PpSKA3-1   61 SRGYNFAIF-------LEDLNDDISSALTELTHLEERTTDTLSFE-----ELLVHCDALY
PpSKA3-2   46 SRDHNFDIF-------LEDLNEDITSALAELQHLEERTTDTLSFE-----ELLVHCDALY
AtSKA3     37 -LDSASSTF-------IKGLNRRISTAASELSFLESMSFGTVSFE-----ELLGHCSQIY
HsSKA3     29 -LDGEESDFEDYPMRILYDLHSEVQTLKDDVNIL----LDKARLENQEGIDFIKATKVLM

PpSKA3-1  109 ESNEDGIRKLELQLQQYGYAP---------DVLHETTYQEM------RTNSHTQDEEISS
PpSKA3-2   94 KSNEDGIAKLELQLQQYGYTPVEVPKQTHIEVLHGHSHQEI------ETKSHILDEAISP
AtSKA3     84 KNNQKDLLHLQDRLTDFGYVP-------EIEIDEGRDEESVFGAFGHEASKHSDDD----
HsSKA3     84 EKNSMDIMKIREYFQKYGYSP-------RVKKNSVHEQEAI-----------NSDPELSN

PpSKA3-1  154 HKSAATPALMNVNETSKVTPTALSYNIK-NAKRDVEYSPLFG-----DLGDLENLESLGI
PpSKA3-2  148 PRSGVTPPSVNENETSITTPVALSYSAK-NTKRDVEYSPLFG-----DLGDLENLESLGI
AtSKA3    133 ---------------------LESHSLQCSIKKGLDEDNL--------LDNSLNLKNLGL
HsSKA3    126 ---------------------CENFQKT-DVKDDLSDPPVASSCISEKSPRSPQLSDFGL

PpSKA3-1  208 SATSLTALTG---------QDEPVSSVHPSLQKLT-FVESPT------------------
PpSKA3-2  202 SATSLTALAS---------QDEPVSSVHPLQHKLT-FVESP-------------------
AtSKA3    164 SDACLAYLATG-------VNDN---------------VKDP-------------------
HsSKA3    164 ERYIVSQVLPNPPQAVNNYKEEPVIVTPPTKQSLVKVLKTPKCALKMDDFECVTPKLEHF

PpSKA3-1  240 ---------NEDARISL---RDAEMEDNLNTKYGSDWFPESKKKAPPPP-----SESDSR
PpSKA3-2  233 ---------KKDVMISM---RDSEIEDNLKTKFGSHRPSEADIKHIPLP-----STSDSM
AtSKA3    183 ---------------------DTSLKESVKGK-------SFDTRALPAP-----NASE--
HsSKA3    224 GISEYTMCLNEDYTMGLKNARNNKSEEAIDTE--SRL--NDNVFATPSPIIQQLEKSDAE

PpSKA3-1  283 LISTQRNAEVCSNPLVHNKPKSEHGDGATN------ATPTTSTPIRDENFQSLDTKKV--
PpSKA3-2  276 FDSTYGHDSI---PVVFNKPVNGCGIGSTN------STTTTTFSTRDETVQSVETNNV--
AtSKA3    208 ------------------------------------------LSNEDE-YATLEMDKTS-
HsSKA3    280 YTNSPLVPTFCTPGLKIPSTKNSIALVSTNYPLSKTNSSSNDLEVEDRTSLVLNSDTCFE

PpSKA3-1  335 ----PSLNEISSVQYDHL---PLWLKSQVSFEEVNAAVTKINELI-RQRGTQGEHANQFF
PpSKA3-2  325 ----PSLTEISSVQYDNL---PIWLRSQVSLQELNGAVAKINDLV-SQRTIGGQHATQFF
AtSKA3    224 ---GPTLTLIKE-EYDSL---PSFMKSLASWEDLLSAVQKFNSVLDSKKEING----SYY
HsSKA3    340 NLTDPSSPTISS--YENLLRTPTPPEVTKIPEDILQLLSKYNSNLATPIAIKAVPPSKRF

PpSKA3-1  387 LDQKDVQSLEL---ARAGIMALTKAEKLVTQFINGVTAYRICVQELVLPP
PpSKA3-2  377 LDQKDVQALGLGSKARAGIMLLTKADKIVTQNINGVTTYRICV-------
AtSKA3    273 FRADEIPTLGLGHKEKAYTLLLTRMKRLVVETTDGVISYRVA--------
HsSKA3    398 LKHG--------------------------QNIRDVSNKEN---------
```

```
PpBorealin    1 MGRPKKAAVV-------RRALLN--KDTNLPAPTEVKPVENVDEEVSDFEEQLRIVNLVC
AtBorealin    1 MPKRKAKECV---------KLTE--EDRNDEKRIRIEEKKE-------------------
HsBorealin    1 MAPRKGSSRVAKTNSLRRRKLASFLKDFDREVEIRIKQIE------SDRQNLLKEVD---

PpBorealin   52 NAVDQEVESRSAAI--------VAIGDGHMA---SATTQLQLM-MSKFPEHIRKMPLKKF
AtBorealin   31 DFVDEEVERQIAAIR--------AIRDVEIE---QMLTALRLL-RSYFTEEQLHTPVLDF
HsBorealin   52 NLYNIEILRLPKALREMNWLDYFALGGNKQALEEAATADLDITEINKLTAEAIQTPLKS-

PpBorealin  100 LKQHCPN-TIANTRSDGTVYFDPVLESEPFLIGRFQAPGGGV-LNFNSLNFNSLNFNTGF
AtBorealin   79 FKENLPDLSISRNEETGEI----ELKWRDENGDSFAGNENGVDMNYSILKRLSMRFTDLY
HsBorealin  111 ----------AKTRK--------VIQVDEMIVEEEEEEENER-KNLQT------------

PpBorealin  158 ASVQNTPGFGPSKTLREQV-GEGVSNEIAGQKDIEQLSWDEFLASYRMQSAMKGPS----
AtBorealin  135 SRSSLGGYDLPDNVKANLL---GTDNP----------QLDNLVFQGTSENQML-------
HsBorealin  140 ARVKRCP---PSKKRTQSIQGKGKGKRSSRANTVTP-------AVGRLEVSMVKPTPGLT

PpBorealin  213 ------AWDTPS--------------LRTPGMNWRDTMITRNYTPM--TEKVFPS-----
AtBorealin  175 PGHD--AFQTPGVNGQRLSFGMTPKTLRLPKAGE--MMLSVHGSPLGVYKE---------
HsBorealin  190 PRFDSRVFKTPG--------------LRTPAAGERIYNISGNGSPLADSKEIFLTVPVGG

PpBorealin  246 -----MLPSQTGKMSVGG------------TKRKAPTSRGLRM--
AtBorealin  222 ------------DHNMGAINEEN-------S--------------
HsBorealin  236 GESLRLLASDLQRHSIAQLDPEALGNIKKLSNRLAQICSSIRTHK
```

```
PpMps1-1     1 MVYPESAMARRD-------------------------PSSEKENMVPRVHRSEMASSGLS
PpMps1-2     1 MVGPESAMARCD-------------------------PSLEKENLVPRIHKPEPGSGGVV
AtMps1       1 -------MDREDNLP--------------------VQPDKPPKSLVRPILNLETTSS---
HsMps1       1 -------MESED-LSGRELTIDSIMNKVRDIKNKFKNEDLTDELSLNKISADTTDNSGTV

PpMps1-1    36 SVRTRGLAASEHRSHAVLKESSLQNQCPAAVNLCKTNLASSDKDWLKNSSADYFSGKQPA
PpMps1-2    36 LTRTRGLTTCEHKSHTALKESSLNNQTPVAATLCKVKFGSTEKGGLKNFADDCYGGKQSG
AtMps1      31 ------------------------------------------------------SSSSSS
HsMps1      53 NQIMMMANNPEDWLSLLLKLE--KNSVPLSDALLNKLIGRYSQA-IEALPPDKYGQNESF

PpMps1-1    96 KSLELLREKIGQLRNGGALKQSSPKTASTLDHHSVGESHSRGHTPSI------------F
PpMps1-2    96 RSLEQLREKLGQLKNVGAQKQTTHKTVSTPEHHS-GELHFRGYTPNA------------F
AtMps1      37 SSPELLRH---------------------------LQAAFKRHRP---------------
HsMps1     110 ARIQVRFAEL--------------KAIQEPDD---ARDYFQMARANCKKFAFVHISFAQF

PpMps1-1   144 E-SSVHVYRD----EKVLNSGGTLKEKMKPESHTRKDEVYSRNISP---PFLEYEKNSIE
PpMps1-2   143 E-SSVHVYKD----EEVSNSGVTPKEVLNAASQGRKRE---KNISSVLTPALGHVESLAE
AtMps1      55 ------------------------LSKMQTTSIGPR-----RSVAP--------------
HsMps1     153 ELSQGNVKKSKQLLQKAVERGAVPLEMLEIA---------LRNLNLQKKQLLSEEE----

PpMps1-1   196 QNYDDRESPVSFSTPSISFTGKGQLFQNKADRGGQITSEKQSSFE----HDMKSIVNQNQ
PpMps1-2   195 DNYDACGKQVSPSTPLMSSMVEGRLVHTKSDSGGVITADKESAISSLAGNEGKCAADHNS
AtMps1      72 ------QRQASRNT---------RLVTAEGQRSQDVVTLSQSLAANTLTQDTT-----NN
HsMps1     200 ------KKNLSAST----------------------VLTAQESFSGSLGHLQNRNNSCDS

PpMps1-1   252 RGENLTA----GETARNTSPQEEVARGSRENSVERLPIPSTIDKI-----IKRAKCSRSG
PpMps1-2   255 QRKTSVA----GETVRNASPREEEAEINHDGSVERR--SAFANKI-----FKRVKGSSYG
AtMps1     112 LAITSVA----GESASITQP-----------TVSEH----------------------FN
HsMps1     232 RGQTTKARFLYGE---NMPPQD--AEIGYRNSLRQT------NKTKQSCPFGRVPVNLLN

PpMps1-1   303 PALRVRRDDPRAYPRTQGPKVDHS-------PSSEKSSDDEQKSAEKL-HLLKTNDNHGN
PpMps1-2   304 PALRVRRDDPRAY-----SKDDHS-------PGSEKSADNVEMSSPKVPHGSTKTDHHSN
AtMps1     135 PSDRQMDFGKSAV-------------------TSLESNLDVQRKSQSL--IGTSQDME--
HsMps1     281 SPDCDVKTDDSVVPCFMKRQTSRSECRDLVVPGSKPSGND----SCELRNLKSVQNSHFK

PpMps1-1   355 DPPVAPAALEA----FNSSNQRMKQDTLMPADEDY---ARKRKAEADPSITDSSLLLSGV
PpMps1-2   352 TQPSAPASSAAGTVSSNKSNTGSNHNVALPTDEDYLNENRKRKADADPSVIDSSLVLTGV
AtMps1     172 ---------------WDATNQ---------AEASHLDACIGSKHQNLPSV-DSE------
HsMps1     337 EPLVSDEKSSELII-TDSITLKNK------TESSLLAKLEETKEYQEPEVPESN------

PpMps1-1   408 GSVRPVSI----EDNEGVHKGGSLTQRERERHQQIINKEIRTDLTSLEQSGERWAEDVAG
PpMps1-2   412 ATDQHSSVVNALEDYESVMKGGKTVERERGRGRGLTVRETGAEFDASEQLGEKWSQDAAG
AtMps1     201 -----VSLKSEYKDSSSLAK----IQGQLGEFPNFLNQPR----TRCSAVGSSWATTTLI
HsMps1     384 -----------------------QKQWQSKRKSECINQNP-------AASSNHWQIPELA

PpMps1-1   464 HPLTGSCTKGTPRYER---------EPWAWKTSRDTRSLV-LPSPTVLD----CTKSAKL
PpMps1-2   472 HPPVVSCAKATTGHDR---------EPWAWKSSRDLRSVA-LTSPNSSE----QLKSAKI
AtMps1     248 HSSSAPMLNATTHVSRSYVEADSNANPHAVQSQGNLPS---------------CCPSSKV
HsMps1     414 RKVNTEQKHTT--FEQPVFSVSKQSPPISTSKWFDPKSICKTPSSNTLDDYMSCFRTPVV

PpMps1-1   510 TGGIPPRYVPPNFESRERKAEANDSPSSEGLKHHCDVLKQMNEAEKREPNNGFSFHSNLN
PpMps1-2   518 IGGLPPRCTPSSLGSRDGKIEANGSPLSNGPKYQSAVLKQVNEAVKSELINGLVPPSTNH
AtMps1     293 SNILHP----------NKDATASEMPAS------------TNDPEVR-------------
HsMps1     472 KNDFPPACQLS---------TPYGQPAC--------------------------------

PpMps1-1   570 VKP-AITDNSDANHLSSTFSSVSIEDRSPRSNLVTKSSMEARTVTQISGHHMNFVATECR
PpMps1-2   578 VKSVASTDDHVVNHLSNSFSSISIGERNA-SGSIWKTSTHARTPTPSSGHQMNSVPPEST
AtMps1     318 VKE---TDTSKQQQIT--------------------------------------------
HsMps1     491 ------FQQQQHQILA--------------------------------------------
```

```
PpBubR1-1    1 -----------------------------------------------MAMNTYENSSSES
PpBubR1-2    1 ----------------------MGTEWELSKENVQPLRKGRKVEILNETLQVKEESPSQS
AtBMF2       1 MAAETKVQVSDPEAEFLNSKQETGYEWELFKENVRPLKRGRNVGILNHALKSHSDHQLRK
AtBMF3       1 MA-----------------KNENGYENLLA------------------------------
HsBubR1      1 MAAVKKEGGALSEAMSL-----EGDEWELSKENVQPLRQGRIMSTLQGALA--QESACNN

PpBubR1-1   14 RDDD-LRRTILNFVSEYSGNDPLEPWLRCIKWLKEMNPPGNVQVVLKEVLELTGNEFLAD
PpBubR1-2   39 KLLDSQRRSMIEAIDAYEGDDPLHPWLQCIRWIKDAYPTGGYQSELLPVVEACTRTFQND
AtBMF2      61 NLIE-KRRNLIEAIDEYEGDDPLSPWIECIKWVQEAFPPGGECSGLLVIYEQCVRKFWHS
AtBMF3      14 --------SLIVDIKSYSGKDHLLPWIRGVKKMKESLPSQILNEKLPRFLQKCAESFESD
HsBubR1     54 TLQQ-QKRAFEYEIRFYTGNDPLDVWDRYISWTEQNYPQGGKESNMSTLLERAVEALQGE

PpBubR1-1   73 NRYKSDLRYLRVWIQYADCCVDRGGIFPFLEANGIGLGHGLFYEAYAMLLESSRDFAKAD
PpBubR1-2   99 ERYKSDIRYLRAWVLYADLCKEPREIYQFLELHCIGQDHALFYEAYATYMELCKKHSKAN
AtBMF2     120 ERYKDDLRYLKVWLEYAEHCADAEVIYKFLEVNEIGKTHAVYYIAYALHIEFKNKVKTAN
AtBMF3      66 KRYKNDSRYIRVWLQLMDFVDDPRALLRTMEAKSIGTKRSLFYQAYALHYEKMKRFEDAE
HsBubR1    113 KRYYSDPRFLNLWLKLGRLCNEPLDMYSYLHNQGIGVSLAQFYISWAEEYEARENFRKAD

PpBubR1-1  133 YVFQLGISRGAQPLERVMLMYNNFRGRMLARQKRKQRQE--------------QELKALL
PpBubR1-2  159 EIYELGLRRDAQPSTRLQNMYQSFLKRMMQR-NERKLQEDQEDAFE---PEKMRHFGD--
AtBMF2     180 EIFNLGISRDAKPVEKLNDAYKKFMVRTMRRSNTADEQEPKENNDL---PS--RSFGTLL
AtBMF3     126 KMYRLGVQNLAEPMDELQKSYLQFVTRMERHKKKKTQRQE-------------QKLSGKH
HsBubR1    173 AIFQEGIQQKAEPLERLQSQHRQFQARVSRQTLLALEKEEEEEVFESSVPQR-STLAELK

PpBubR1-1  179 ARG-----AQRPLSGSNGI-----QGLPTMPPF-QNSAREDPLPDMQVSVGG----YNGP
PpBubR1-2  213 SRGPPTRAPMRPSFG-HDQ-----QRRKMHQP-------QVPRSTMDIFVD--DEFHSGT
AtBMF2     235 SRG--DNNARRQALGSSNP-----QAKKLKPN-------QSSKTPFAIYADAVSDTTSGN
AtBMF3     173 HKV---------------------DERQQHEPVLN-------------FVDK----AIVG
HsBubR1    232 SKG--KKTARAPIIRVGGALKAPSQNRGLQNPFPQQMQNNSRITVFDENADE-ASTAELS

PpBubR1-1  224 APVLIGSKIYESTAVKAESSFEEERMRHWVHNKSSQLRSATYNAPVAFNNVQNMSNSIHT
PpBubR1-2  258 RP-----------SVVPEPV--------GVH--------------VPAATWKNLG-----
AtBMF2     281 QP--------ESDKSRPE-----------------------------FGSWLMLG-----
AtBMF3     195 KP--EAENACHHGLVDPTINMKE--AMNTINN--------MFKEPIETAPLQRRS-----
HsBubR1    289 KP-----------TVQP-----------WIA------------PPM--------------

PpBubR1-1  284 DTRTRVNEVNHNPYRPGIRMDSMPNQMPGQKTETSNGFRNSVPKLSQSVRSSQPADAG--
PpBubR1-2  280 -TQKEVRKEN--------------DQRPSKWNET------TLPRLGK-VSKHVEARPP-I
AtBMF2     299 -GRAERNKEN--------------NSLPRKWASF------KVPQKPI-VRTVAAASASTF
AtBMF3     238 -RQRSQNKENQGCNNS-------------------------------------------F
HsBubR1    301 -PRAKENELQAGPWNTGRSL----EHRPRGNTAS----LIAVPAVLPSFTPYVEETAR--

PpBubR1-1  342 NVLSNDSFGGEKHLTRPATSSFVTAVQQHRSL----------------PLPHRDLPQTGR
PpBubR1-2  317 EVFVD-EECEEAHARLKVKNASASSTTLRQRVDGVCDLRREQEALQKNPLMHFN---DGE
AtBMF2     337 EVFVDEEECTEEEEEKKKNDETISSSSNVLPLNGGREIKKETELLRQNPLRHF-------
AtBMF3     254 EVFLDENLECETGTSGKAKTSTT---------------------------------QCGS
HsBubR1    350 -------------------QPVMTPCKIEPSINHILSTRKPGKE-EGDPLQRVQSHQQAS

PpBubR1-1  386 SHTNENLKGMERNGNLAEPAGHSENIDDVTIVTKSVTDDIMAMFADSRPASLHSETTVR-
PpBubR1-2  373 KPSTASLLPRDPSGNSREPDGPESGAPHL------VGSEIFQGGEDGRETCFEEARLVRW
AtBMF2     390 ------------------------------------------------------------
AtBMF3     281 QPNQESFE----------------------------------------------------
HsBubR1    390 EEKKEKMM--------------------------YCKEKIYAGVG---EFSFEE---IR-

PpBubR1-1  445 ---PPSAHENSFKTERSYTNENVRGMERNCSSGERAGQSKIVDDATIMTKSVTDDIMAMF
PpBubR1-2  427 KMPHPAAHHPAVSSPR--------------SSAEKA---KLIEKS-----------LNMF
AtBMF2     390 ------------------------------------------------------------
AtBMF3     289 ---------------------------------------IFIDDE---------------
HsBubR1    417 ----AEVFRKKLKEQRE--------AELLTSAEKRAEMQKQIEEMEKKLKEI--------

PpBubR1-1  502 SDPRETSSRSEAVAQEYASPLASHRGMPKIEKSYTNENLAGTVVNVSSVQRPGQSEVLDD
PpBubR1-2  459 --------QSKNAKQKNQDSRFSDREVPVPERRVHDIDISNKM--VGKLSRGSSNE----
AtBMF2     390 ------------------------------------------------------------
AtBMF3     295 -------NTDETADENDEAGKAFVFLLPRDHS-------------------PESSE----
HsBubR1    457 ----QTTQQERTGDQQEET-------MPTKE--------------TTKLQIASESQKIPG
```

```
PpBubR1-1  562 LTMDSKSVTDDIMSMFSDSFA----RKEPGQRGVTSSFA-----------KTTVPYAGTQ
PpBubR1-2  505 ------SSSDESVASFLAPPA----TKSSMSRASSMSSI-----------DEPPPFACSM
AtBMF2     390 ------------------------------------------------------------
AtBMF3     325 -----------------------------------------------EADRNTPP-----
HsBubR1    492 MTLSS-SVCQVNCCARETSLAENIWQEQPHSKGPSVPFSIFDEFLLSEKKNKSPP-----

PpBubR1-1  607 KGTPPVNGGLRAPVAPNQPVRKSANASAPHNSPSTSIPSDSESRTLNVAADYKGITASIQ
PpBubR1-2  544 --------------------------------PNSQFTANGEE------------TIAIK
AtBMF2     390 ------------------------------------------------------------
AtBMF3     333 ----------RARFREDTVVRRF---------VGSTISDEPEA-----------------
HsBubR1    546 -ADPPRVLAQRRPLA----VLK----------TSESITSNED-----VSPDVCDEFTGIE

PpBubR1-1  667 QQSSHIAVGGKSMAAEGVKPEASAHTSWSSDNQVHNKAATIHPR-----EFVSVHKDVAS
PpBubR1-2  560 KYAEELIIHGAD--PNGRRTDVGRHQGLIDQ--------TINTK-----ECL---QDILS
AtBMF2     390 ------------------------------------------------------------
AtBMF3     357 ------------------VENACHHGLVDP---------TVNLK-----EAM---EDINN
HsBubR1    586 PLSEDAIITGFR-------------------------NVTICPNPEDTCDFARAARFVST

PpBubR1-1  722 SSTSAVDTEAPPNQKSMEQTAVGLRITKESRGSIPDEHSKPDQGTAQGRSCGKNRVNNEY
PpBubR1-2  602 MFNRPLSCEKVPQRKP--------RASKSSR---------PSPNSTEG------------
AtBMF2     390 ------------------------------------------------------------
AtBMF3     382 MFGEPINFVRPNRSKNKG------KAVVETK-----------PNPAAG------------
HsBubR1    621 PFHEIMSLKDLPSDPE--------RLLPEED---LDVKTSEDQQTACG------------

PpBubR1-1  782 SVYDDS-SIRSTSSRKRSSWSTSAKGSTHCIYLSDEEEK--IDNRDNKKQKVQVASTTFN
PpBubR1-2  633 --FD--------------------------VFIDDDEDNPPVPRPQNNGVNLNRGFEIFN
AtBMF2     390 ------------------------------------------------------------
AtBMF3     413 --FS--------------------------ILEDDDEEE---------------------
HsBubR1    658 TIYSQTLSIKKLS-----------------PIIEDSREA--------------------T

PpBubR1-1  839 EGSGVRESGTPVMDRSDVTREQQPSQYSPI-------------VCSTPSKS---------
PpBubR1-2  665 D------------------EENDPSQLSKIHAKKGFEVFVDEVISAPPPRT------RKL
AtBMF2     390 -----------------------------------------------PP-----------
AtBMF3     424 -------------------AEQEHQGKSQP-------------TQRLPSKS---------
HsBubR1    681 HSSGFSGSSASVASTSSIKCLQIPEKLELTNE-----------TSENPTQSPWCSQYRRQ

PpBubR1-1  877 -ITTF--------------KGS-TSLQRNILEPTLSRTEHRKKPG-GQLYPWEQSLLASL
PpBubR1-2  701 KMNSFEVFQDVPDVVKQPGKGSFEVFEDEELPP--------KKPGRGSFEIFEDEL----
AtBMF2     392 --NSF-------------------------------------------------------
AtBMF3     443 ------------D-------------ERELFEPTVC------------------------
HsBubR1    730 LLKSL---------------------------PELSAS-------------------AEL

PpBubR1-1  920 LKELDPPLSKYKGYIYSSKKYSGKVSLATFAS----TKPG------VKNKPLELGCSKYQ
PpBubR1-2  749 ------PLER-----------PGKGSFEVFEDELPPTKPGKGSFEVFQDEPASTKAAK--
AtBMF2     395 ------------------------------------------------------------
AtBMF3     454 ------------------------------------TKVA--------------------
HsBubR1    744 CIE-DRPMPK-----------------------------------LEIEKEIELGNEDYC

PpBubR1-1  970 LKGCTGRGAFAQVYLADALHN------NGDSHRVVLKVQKPPCPWEFYIYRQLDVRILSE
PpBubR1-2  790 -------GNF----LVDEV-----------KHRVA----RPP------------------
AtBMF2     395 ------------------------------------------------------------
AtBMF3     458 ---------------LDEINKLFAM------------------PMDF-------------
HsBubR1    768 IK---------REYLICEDYKLFWVAPRNSAELTVIKVSSQPVPWDFYINLKLKER-LNE

PpBubR1-1 1024 E---RCSYGCAQN------IHIYADCSFMECDFGQYGTLQDVINSYLSKGQRMDEALCIF
PpBubR1-2  806 -------------------------------------------------ARKIPSAMC--
AtBMF2     395 ------------------------------------------------------------
AtBMF3         ------------------------------------------------------------
HsBubR1    818 DFDHFCS--CYQYQDGCIVWHQYINCF----------TLQDL----LQHSEYITHEITVL

PpBubR1-1 1075 YTIEMLHMLENLHKVGLIHGDFK---PDNLLIRSSSENLGVWTPDKSGSWKQQGVCLIDW
PpBubR1-2  815 ------------------EGSFDIFVDDDVVAKSS-------VPKQGSS-----------
AtBMF2     395 ------------------------------------------------------------
AtBMF3         ------------------------------------------------------------
HsBubR1    862 IIYNLLTIVEMLHKAEIVHGDLS---PRCLILRNR-----IHDPYDCNK-NNQALKIVDF
```

```
PpBubR1-1 1132 GRSIDL-------TLFAEGTEFVGDSKTDGFRCTEMIEKK------PWTFQVDTYGLCGV
PpBubR1-2  839 ----------------SRGTE--------------------------STFQV--------
AtBMF2     395 ------------------------------------------------------------
AtBMF3         ------------------------------------------------------------
HsBubR1    913 SYSVDLRVQLDVFTL-------------SGFRTVQILEGQKILANCSSPYQVDLFGIADL

PpBubR1-1 1179 AHCLLHGNYMEIDKRSSN-GAPQVVYRPKAPYKRYWNVNLWQNFFETLLNLKTDKSYDTL
PpBubR1-2  849 --------YADED---SI-PAPKNVER------------------------RIPKSKPVS
AtBMF2     395 ------------------------------------------------------------
AtBMF3         ------------------------------------------------------------
HsBubR1    960 AHLLLFKEHLQVFWDGSFWKLSQNISELK-------DGELWNKFFVRILNANDEATVSVL

PpBubR1-1 1238 NKLRRSFEDYLCSDSALTKKLRDLLTKQNTM-----LLSRK
PpBubR1-2  873 KSQTGGFSIFIDDDVAV-QPVRKPKLQSST---------RK
AtBMF2     395 --------------------LR-------------------
AtBMF3         -----------------------------------------
HsBubR1   1013 GELAAEMNGVF--DTTFQSHLNKALWKVGKLTSPGALLFQ-
```

```
PpMAD2     1 MASK-QPTRDVITLKGSAAIVSEFLCYSVNSILFQRGVYPADEFGRVKKYGMTLLMCQQE
AtMAD2     1 MASKTAAAKDIITLHGSAAIVSEFFCYAANSILYNRAVYPEESFVKVKKYGLPMLLIEDE
HsMAD2     1 MALQ-LSREQGITLRGSAEIVAEFFSFGINSILYQRGIYPSETFTRVQKYGLTLLVTTDL

PpMAD2    60 RVKAFIDTITQQIATWLETGTLQRVVMVIASMSTKEVLERWNFNIENDNEVIEKGVTREK
AtMAD2    61 SVKSFMSNLTSQISEWLEAGKLQRVVLVIMSKATGEVLERWNFRIETDNEVVDKGVSREK
HsMAD2    60 ELIKYLNNVVEQLKDWLYKCSVQKLVVVISNIESGEVLERWQFDIECDKTAKDDSAPREK

PpMAD2   120 SDKDIMTEIQAIMRQIAASVTFLPNIEEACTFDLLAYTSVDSEVPAEWAESDARLIHNPQ
AtMAD2   121 SDKEIMREIQAIMRQVASSVTYLPCLDETCVFDVLAYTDTDVAVPFTWIESDPKLIANPQ
HsMAD2   120 SQKAIQDEIRSVIRQITATVTFLPLLEVSCSFDLLIYTDKDLVVPEKWEESGPQFITNSE

PpMAD2   180 VVKLRSIDTMVHQVEAMVAYK------FTEEL
AtMAD2   181 MVKLHGFDTKIHKVDTLVSYK---NDEWDEEE
HsMAD2   180 EVRLRSFTTTIHKVNSMVAYKIPVND------
```
